# TREM2 as a central immunometabolic hub in Alzheimer’s disease: linking microglial states, lipid stress, and disease-stage-specific intervention

**DOI:** 10.3389/fimmu.2026.1893545

**Published:** 2026-07-31

**Authors:** Guang Yang, Xiao Xu, Ying Xu

**Affiliations:** 1 Department of Physiology, School of Integrative Medicine, Shanghai University of Traditional Chinese Medicine, Shanghai, China; 2 Shanghai Institute of Traditional Chinese Medicine for mental health, Shanghai, China

**Keywords:** Alzheimer’s disease, lipid metabolism, microglia, therapeutic targeting, TREM2

## Abstract

Triggering receptor expressed on myeloid cells 2 (TREM2) plays a crucial role in regulating microglial function in Alzheimer’s disease (AD) and other neurodegenerative disorders. Genetic studies have identified rare coding variants in TREM2 as significant risk factors for late-onset AD (LOAD), highlighting the importance of disrupted microglial signaling in disease pathogenesis. Biochemically, TREM2 acts as a receptor for lipid- and damage-associated molecular patterns, recognizing anionic phospholipids, myelin-derived lipids, apolipoproteins, and aggregated amyloid-β (Aβ). It engages with adaptors like DAP12 and DAP10, activating key signaling pathways, including SYK, PI3K-AKT-mTOR, and PLCγ2, leading to microglial transcriptional and metabolic reprogramming. These processes are essential for the transition of microglia to disease-associated microglia (DAM), influencing amyloid plaque compaction and tau pathology propagation. This review aims to synthesize the latest insights into TREM2 biology, focusing on the role of TREM2 in microglial state transitions, lipid metabolism, and myelin turnover. It also examines the pathophysiological relevance of soluble TREM2 (sTREM2) and AD-associated TREM2 variants. Furthermore, the review explores the therapeutic potential of targeting TREM2, including strategies based on agonistic antibodies and modulation of receptor shedding. Beyond prior descriptive summaries, we organize these findings within a stage-resolved immunometabolic framework that links disease timing, lipid-stress context, and microglial state transitions. This framework is intended to explain why similar TREM2-directed interventions may yield different outcomes across disease stages and pathology compositions. We further highlight stage-specific translational logic, including biomarker-informed (e.g., sTREM2-guided) stratification and monitoring, to support testable and clinically actionable trial designs.

## 1 Introduction

Alzheimer’s disease (AD) is the most prevalent cause of dementia worldwide and is characterized histopathologically by extracellular amyloid-β (Aβ) plaques, intracellular neurofibrillary tangles composed of hyperphosphorylated tau, synaptic loss, and progressive neurodegeneration in selectively vulnerable brain regions (1–3). For decades, the amyloid cascade hypothesis has provided a conceptual framework in which Aβ accumulation is considered the initiating event that ultimately leads to tau pathology, synaptic failure, and cognitive decline (4–6). However, longitudinal biomarker studies now indicate that amyloid deposition, tau spread, and neuroinflammation evolve over many years in partially independent trajectories, and the limited success of anti-amyloid monotherapies together with the heterogeneity of clinical courses have motivated a broader view that incorporates vascular, metabolic, and immune mechanisms as integral components of AD pathogenesis (7–11).

Among these additional components, neuroinflammation has gained particular attention (12–14). Microglia, the resident innate immune cells of the central nervous system (CNS), were long regarded as bystanders that react passively to neuronal death and protein aggregation (15–18). Recent work has fundamentally revised this view. Large-scale transcriptomic profiling, including single-cell and single-nucleus RNA sequencing in both mouse models and human tissue, has revealed a rich spectrum of microglial states that are dynamically regulated across development, aging, and disease, and that are tightly intertwined with AD risk and progression (19–22). Developmental and homeostatic programs maintained by signals such as transforming growth factor beta (TGF-β) and colony stimulating factor 1 receptor (CSF1R) help preserve the adult microglial phenotype (15, 23). Homeostatic microglia support neuronal and myelin integrity and express canonical markers such as purinergic receptor P2Y12 (P2RY12), transmembrane protein 119 (TMEM119) and chemokine receptor 3 (CX3CR1) (15, 24). Once pathological protein assemblies and tissue stress arise, this homeostatic network becomes destabilized and microglia transition into distinct reactive states, suggesting that the way microglia lose their homeostatic identity and gain disease-associated features is itself a critical determinant of AD outcome (21, 22, 25).

Genetic studies have firmly placed microglia at the center of LOAD pathobiology. Genome-wide association studies (GWAS), whole-exome and whole-genome sequencing have identified numerous AD risk loci enriched in microglia-expressed genes, including triggering receptor expressed on myeloid cells 2 (*TREM2*), apolipoprotein E (*APOE*), cluster of differentiation 33 (*CD33*), complement receptor 1 (*CR1*), ATP-binding cassette subfamily A member 7 (*ABCA7*), inositol polyphosphate-5-phosphatase D (*INPP5D*), phospholipase C gamma 2 (*PLCG2*) and ABL interactor 3 (*ABI3*), implicating innate immune pathways as key determinants of disease susceptibility and resilience (26–28). Many of these risk alleles either alter amino-acid sequence in microglial receptors and signaling molecules or affect regulatory elements that shape microglial transcriptional programs. The convergence of AD risk at microglial genes argues that altered microglial sensing and response may contribute directly to pathogenesis in at least some disease contexts (28–31). Within this network of risk genes, *TREM2* has emerged as particularly compelling. Rare missense variants such as R47H in *TREM2* confer a two- to four-fold increased risk of LOAD, comparable to the effect size of one copy of APOE ϵ4, and homozygous loss-of-function mutations in TREM2 or its signaling adaptor DNAX activation protein 12 (DAP12, also known as TYROBP or KARAP), cause Nasu-Hakola disease (PLOSL), a rare early-onset dementia associated with bone cysts and sclerosing leukoencephalopathy (32, 33). Population studies further indicate ancestry-dependent heterogeneity in TREM2 risk variants, underscoring the context dependence of TREM2-related vulnerability (34, 35). TREM2 variants are also implicated in other neurodegenerative syndromes, highlighting a broader role in microglial dysfunction across diseases (36, 37).

Parallel work at the molecular and cellular levels has begun to explain why TREM2 is so influential. TREM2 is a type I transmembrane receptor of the immunoglobulin superfamily whose extracellular V-set domain recognizes a remarkably broad repertoire of ligands (28, 29), including anionic phospholipids exposed on stressed or apoptotic cells (38, 39), myelin-derived lipids (40), lipoproteins and apolipoproteins such as APOE and clusterin (CLU)/apolipoprotein J (APOJ) (41–43), and aggregated or lipid-associated Aβ (44, 45). Ligand binding engages canonical adaptor-dependent signaling that regulates microglial survival, phagocytosis, and metabolic adaptation (29, 46–51). These signaling properties situate TREM2 at the interface between microglial activation and lipid metabolism. Several reviews have outlined TREM2 as a lipid sensor that is particularly important in environments with high lipid flux, such as amyloid plaques, demyelinating lesions and metabolically stressed peripheral tissues (37, 50). In AD and demyelination models, TREM2 is required for full acquisition of plaque- and damage-associated microglial states and for effective lipid handling, including cholesterol processing and myelin debris clearance (20–22, 37, 52–55). Together, these findings identify TREM2 as a key coordinator of microglial responses to lipid-rich damage in the CNS.

Another layer of complexity arises from the proteolytic processing of TREM2. Sequential cleavage by A disintegrin and metalloproteinase 10/17 (ADAM10/17) and γ-secretase generates soluble TREM2 (sTREM2), comprising the extracellular ectodomain, which can be detected in cerebrospinal fluid and blood (56, 57). CSF sTREM2 increases during preclinical and prodromal AD and is associated with tau-related and neurodegenerative markers (58, 59). sTREM2 may also exert signaling functions distinct from membrane-bound TREM2 (60, 61).

Importantly, the role of TREM2 is not restricted to the CNS. TREM2-expressing macrophage populations have been identified in adipose tissue, liver and tumor microenvironments, where they respond to obesity-related metabolic stress, hepatosteatosis or cancer-associated inflammation by adopting specialized lipid-associated or immunosuppressive phenotypes (29, 62). The fact that the same receptor is deployed in such diverse pathological conditions underscores that TREM2 senses generic features of tissue damage and lipid overload, while the downstream consequences of its activation are highly context-dependent (50). For AD, this cross-disease perspective is informative: manipulations of TREM2 that are neuroprotective in a degenerating brain might be detrimental in a tumor setting, and vice versa, emphasizing the need for nuanced therapeutic strategies.

Taken together, these genetic, molecular and cellular observations indicate that TREM2 is a central node connecting microglial biology, lipid metabolism and neurodegenerative disease. However, existing studies and reviews have often addressed these themes in isolation, with limited integration of their conceptual and mechanistic interconnections (21, 28, 29).

**Figure 1:**
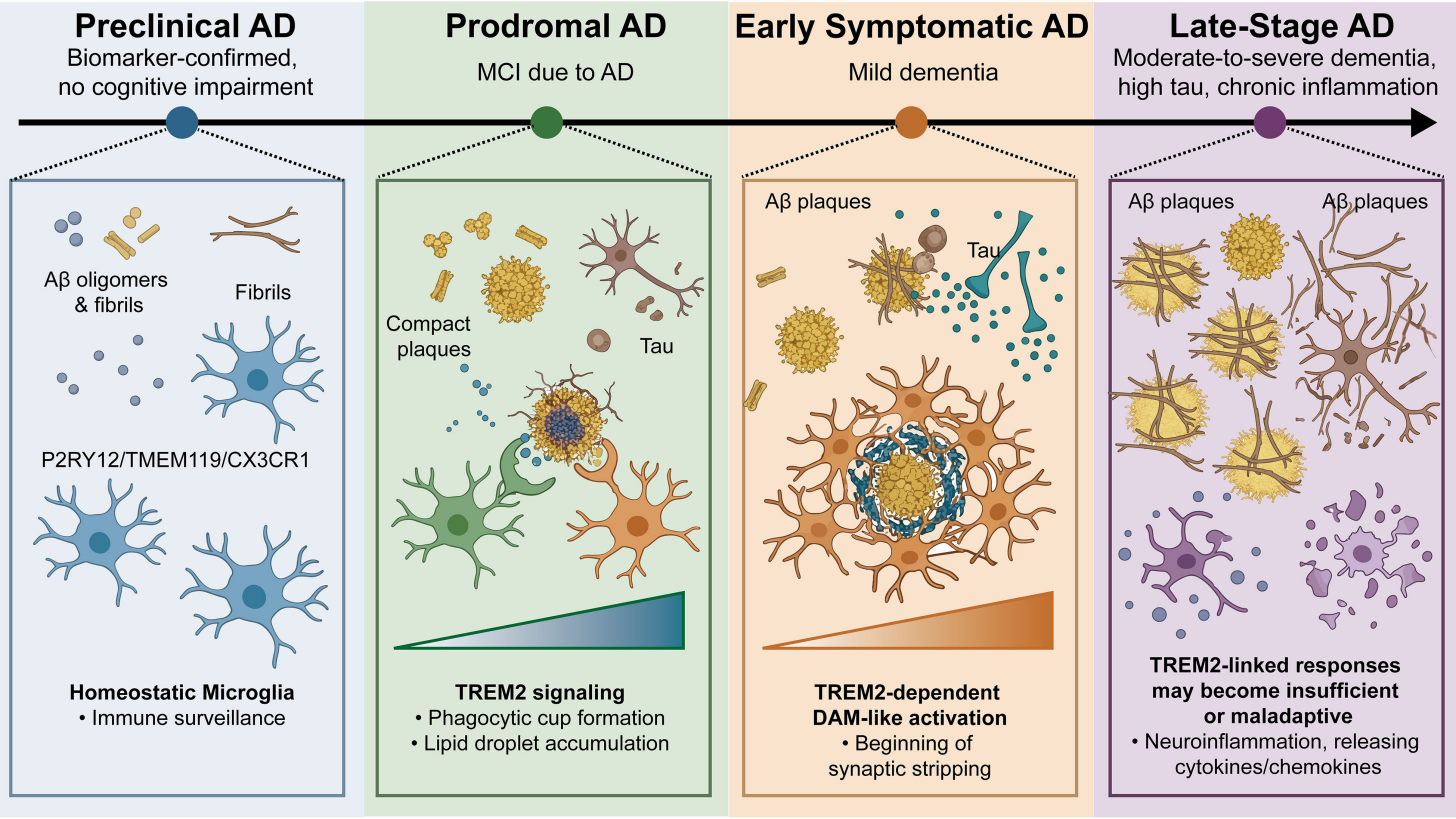
Operational AD stages and stage-specific TREM2-microglial responses. Schematic summary of the operational AD continuum, including preclinical AD (biomarker-confirmed, no cognitive impairment), prodromal AD (mild cognitive impairment due to AD), early symptomatic AD (mild dementia), and late-stage AD (moderate-to-severe dementia with prominent tau pathology and chronic neuroinflammation). Across disease progression, microglia transition from homeostatic states toward TREM2-dependent DAM-like phenotypes and, in advanced disease, may exhibit insufficient or maladaptive TREM2-linked responses.

Recent reviews have substantially advanced the TREM2 field by summarizing receptor biology, ligand recognition, signaling pathways, genetic risk, and therapeutic development (21, 28, 29, 50, 63). Here, we build on these contributions by integrating these dimensions within a stage-resolved immunometabolic framework. Specifically, we examine how TREM2 links extracellular damage sensing to lipid metabolism, microglial state transitions, soluble TREM2 biology, and pathological outcomes across disease stages, while highlighting unresolved issues such as ligand hierarchy *in vivo*, ancestry-dependent variant heterogeneity, and the boundary between protective and maladaptive TREM2-driven responses. This framework is intended to clarify why similar TREM2-directed interventions may yield different effects depending on timing, pathology composition, and host context. A structured comparison with representative recent reviews is provided in **Table 1**, and disease-stage staging is summarized in **Figure 1**.

**Table 1:** Differentiation of the present review from representative recent TREM2 reviews (2023-2026).

Dimension	Zhao et al., (28) (Ageing Res Rev, 10.1016/j.arr.2024.102596)	Wang et al., (123) (Front Immunol, 10.3389/fimmu.2026.1739875)	Present review
Core framing	TREM2 as a bridge between microglia and extracellular microenvironment	TREM2-centered neuroimmune integration across mechanisms, genetics, and therapy	TREM2 as a stage-resolved immunometabolic hub
Microglial states	DAM development and AD-related activation states summarized	DAM/TREM2-APOE axis and state changes discussed	State transitions mapped to lipid stress context and disease timing
Lipid metabolism integration	Included, but not the primary organizing axis	Included (APOE/lipid crosstalk emphasized)	Central organizing axis: cholesterol/myelin/lipoprotein burden constraining microglial responses
Temporal (disease-stage) resolution	Therapeutic discussion present, limited stage-explicit decision logic	Stage-dependent dual effects acknowledged	Explicit intervention windows and risk–benefit shifts across progression
sTREM2 treatment	Mechanistic and biomarker relevance discussed	Biomarker and therapeutic relevance discussed	Mechanism-to-translation bridge: sTREM2 for staging, monitoring, and trial enrichment hypotheses
Contradictions handling	Controversies discussed narratively	Integrative synthesis with broad scope	Structured “established vs contradictory vs testable next” framework
Translational output	Therapeutic prospects overview	Immunotherapeutic opportunities overview	Decision-oriented framework for stratified, stage-specific TREM2 modulation
Distinct added value	Strong mechanistic landscape	Broad genetics-to-therapy synthesis	Framework-level integration with testable translational implications

In this review, we focus on the role of TREM2 in AD pathogenesis by bringing together its structural and biochemical properties, developmental and homeostatic functions, microglial state transitions, lipid and myelin metabolism, and genetic evidence from human disease. We further discuss TREM2 as a therapeutic target and highlight how context-dependent features of TREM2 signaling, such as disease stage, ligand milieu and peripheral involvement, may define temporal and spatial windows during which modulating this pathway is beneficial.

## 2 Molecular structure and ligand recognition properties of TREM2

**Figure 2:**
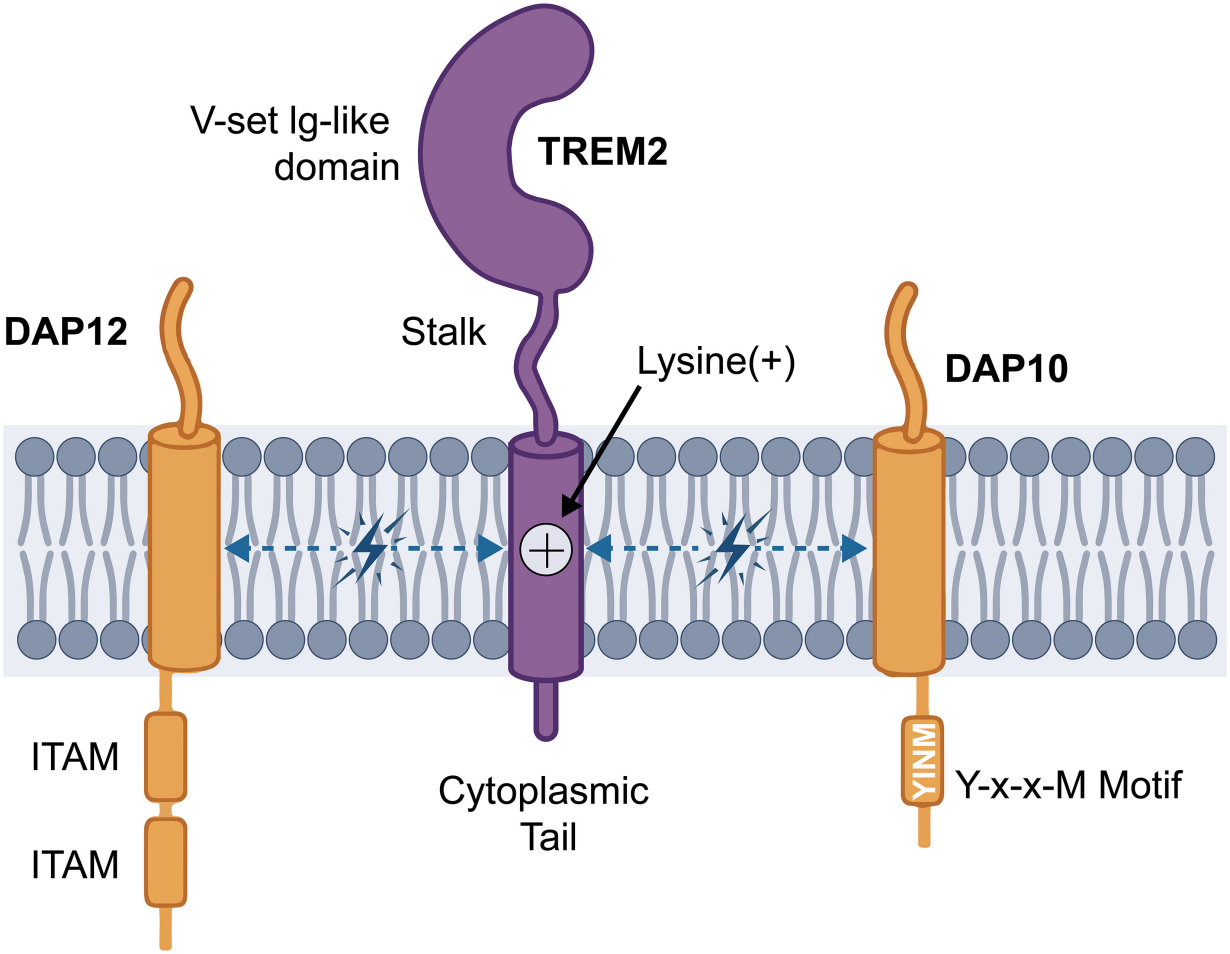
Structural organization and signaling of the microglial TREM2 receptor complex. Schematic representation of TREM2 as a type I membrane receptor on the microglial plasma membrane. The extracellular region comprises a single V-set Ig domain stabilized by β-strand architecture and a flexible electrostatic surface, depicted interacting with structurally diverse ligands. The Ig domain is linked to the membrane by a short stalk and a single transmembrane helix containing a positively charged lysine residue, which mediates association with the ITAM-bearing adaptor DAP12 and the YINM-containing adaptor DAP10 embedded in the membrane.

A central mechanistic role of TREM2 in Alzheimer’s disease is to drive the reprogramming of microglia from homeostatic surveillance states toward disease-associated phenotypes with distinct functional and metabolic properties. Structurally, this broad sensing capacity is conferred by a single extracellular V-set Ig domain linked to a short stalk, a transmembrane helix, and a minimal cytoplasmic tail (29). The receptor is encoded within the TREM locus on chromosome 6p21, a genomic cluster enriched for immunoglobulin-superfamily genes involved in innate immune recognition (64). Its extracellular region consists of a single V-set Ig domain that is stabilized by conserved β-strand architecture yet displays substantial electrostatic flexibility, enabling interactions with structurally diverse ligands (65). This domain is tethered to the membrane by a short stalk, followed by a transmembrane helix containing a positively charged lysine residue that anchors TREM2 to adaptor proteins, and finally a minimal cytoplasmic tail incapable of autonomous signaling (29) (**Figure 2**). Because its cytoplasmic tail lacks intrinsic signaling capacity, TREM2 depends on adaptor proteins such as DAP12 and DAP10 for downstream signaling (47, 48).

Accumulating structural and biochemical evidence reveals that the TREM2 Ig domain is finely tuned to recognize molecular signatures associated with cellular distress, lipid imbalance, and extracellular aggregation (29). At the core of its ligand-binding surface lies a basic electropositive patch that accommodates negatively charged or zwitterionic headgroups, permitting detection of phosphatidylserine exposed on apoptotic membranes, cardiolipin released during mitochondrial damage, and multiple phosphoinositides enriched at sites of membrane turnover. These features help explain TREM2 recognition of lipid-rich debris that accumulates during demyelination and tissue injury, as well as its responsiveness to lipoprotein-associated signals involved in lipid transport and clearance (42, 55). The convergence of ligand classes associated with lipid transport, cellular injury, and debris clearance underscores the concept of TREM2 as a broad-spectrum sensor linking metabolic stress to microglial activation (63).

Importantly, structural studies further suggest that TREM2 preferentially recognizes Aβ in lipid-associated assemblies rather than as an isolated peptide species (66). Although Aβ does not possess canonical lipid headgroups, it often accumulates in a lipid-rich extracellular milieu, where it associates with apolipoproteins and membrane fragments (67). Structural studies show that TREM2 preferentially binds Aβ species complexed with these lipid cofactors, suggesting that the receptor is not acting as a classical protein-protein binding partner but rather as a detector of composite damage-associated molecular patterns (45). This explains why TREM2-dependent microglial recruitment to plaques is tightly coupled to plaque maturation and the deposition of lipidated fibrillar structures (68).

The profound functional impact of AD-associated TREM2 variants further highlights the importance of its ligand interface (33). The R47H substitution, which lies adjacent to the positively charged binding patch, weakens interactions with phospholipids, apolipoproteins, and lipid-Aβ complexes (33). As a result, downstream signaling through SYK and PI3K is attenuated, limiting the metabolic reprogramming necessary for the transition from homeostatic microglia to DAM-like states (69). Variants such as H157Y accelerate proteolytic cleavage, reducing membrane TREM2 and shifting signaling balance toward soluble forms (35, 70, 71).

Together, these structural and functional attributes position TREM2 as a molecular integrator at the interface of lipid biology, tissue damage, and innate immune surveillance. Rather than acting as a receptor for a single canonical ligand, TREM2 detects a constellation of lipid-associated cues generated during myelin breakdown, neuronal injury, and extracellular protein aggregation. This broad recognition logic allows microglia to convert diverse biochemical disturbances into coordinated activation programs, enabling containment of pathology but also rendering the system vulnerable to perturbation through disease-associated mutations. Given the centrality of these interactions to microglial phenotype transitions in AD, detailed dissection of TREM2-ligand dynamics remains pivotal for understanding how microglia interpret pathological signals and for identifying therapeutic windows in which targeted modulation of TREM2 might restore protective functions.

## 3 TREM2-associated signaling pathways

**Figure 3:**
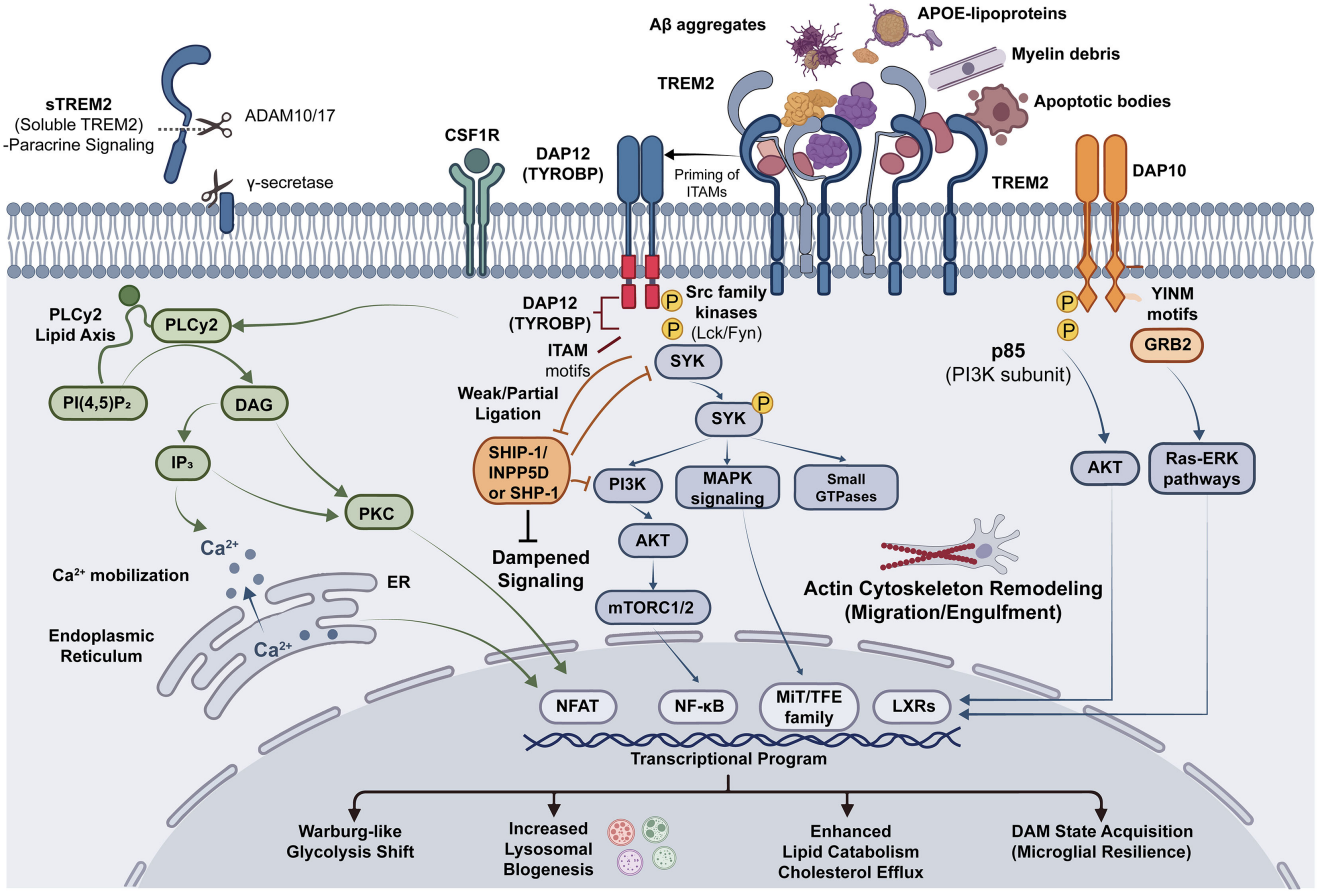
TREM2 signaling nexus in microglia integrating lipid sensing with metabolic and functional reprogramming. Ligand engagement induces clustering of TREM2 with ITAM-bearing DAP12 and YINM-containing DAP10 at the microglial plasma membrane. This clustering promotes Src family kinase-mediated phosphorylation of adaptor motifs, leading to recruitment of SYK or the p85/GRB2 complex. Downstream, multiple signaling pathways are activated: PI3K-AKT-mTOR, MAPK, small GTPases, and PLCγ2. CSF1R signaling can prime DAP12 ITAMs, lowering the threshold for TREM2 responses. SYK- and PLCγ2-dependent hydrolysis of PI (4, 5) P2 generates DAG and IP3, coupling TREM2 engagement to intracellular Ca^2+^ mobilization, PKC activation, and transcriptional programs via NFAT and NF-κB. These events collectively drive Warburg-like glycolysis, lysosomal biogenesis, lipid catabolism, and disease-associated microglia (DAM)-like states. Negative regulatory mechanisms are indicated, including ITAMi-mediated recruitment of SHP-1 and SHIP-1/INPP5D-mediated hydrolysis of PIP3, which constrain PI3K-AKT signaling and modulate TREM2-dependent microglial functions. Proteolytic shedding of TREM2 generates sTREM2, which can act in a paracrine manner to influence neighboring microglia. Together, these pathways illustrate how TREM2 signaling balances activation and inhibition to control microglial metabolic and functional states.

TREM2 couples an unusually diverse set of extracellular danger and lipid cues to a relatively compact intracellular signaling module (50). Because its cytoplasmic tail is extremely short and lacks canonical ITAM or ITIM motifs, the receptor is entirely dependent on membrane adaptors to translate ligand binding into downstream responses. At the structural level, this coupling is mediated by charge-charge interactions between a conserved lysine in the TREM2 transmembrane helix and acidic residues in the transmembrane segments of DAP12 and DAP10 (48) (**Figure 3**). Depending on cell type and context, TREM2 can associate with DAP12 alone, DAP10 alone, or mixed TREM2-DAP12-DAP10 complexes, suggesting that the stoichiometry and composition of these assemblies already encode a first layer of signaling diversity (48).

Ligand engagement induces clustering of TREM2-adaptor complexes within the plasma membrane, bringing multiple ITAM motifs into proximity with Src family kinases that phosphorylate the paired tyrosines of DAP12. Phosphorylated ITAMs recruit SYK, which in turn initiates several canonical myeloid signaling cascades, including PI3K-protein kinase B (AKT)-mammalian target of rapamycin (mTOR), mitogen-activated protein kinase (MAPK) modules, and small GTPase networks that remodel the actin cytoskeleton (49) (**Figure 3**). In microglia, this axis enhances survival signaling, supports migration and process remodeling around plaques, and licenses efficient engulfment of apoptotic bodies, myelin debris, and Aβ-lipoprotein complexes (51). In parallel, TREM2-associated DAP10 can directly recruit the p85 regulatory subunit of class I PI3K and adaptor proteins such as growth factor receptor-bound protein 2 (GRB2), providing a SYK-independent route to AKT and Ras-extracellular regulated protein kinases (ERK) activation (48). This dual access to PI3K-AKT signaling may help explain why TREM2-deficient microglia develop lipid droplet accumulation and metabolic exhaustion under chronic stress (51). In particular, SHIP-1/INPP5D negatively regulates the DAP12-coupled PI3K pathway downstream of TREM2 by hydrolyzing PIP3, thereby limiting AKT signaling output and modulating TREM2-dependent programs linked to microglial survival, phagocytosis, metabolic fitness, and inflammatory responsiveness. As INPP5D is also an AD risk gene identified in GWAS, this regulatory node is of particular relevance to disease-associated microglial dysfunction (48, 72) (**Figure 3**).

A second major branch of the pathway is built around phospholipase C gamma 2 (PLCγ2), which is recruited to phosphorylated DAP12 and activated in a SYK-dependent manner (46). PLCγ2 hydrolyzes PI (4, 5)P_2_ into diacylglycerol (DAG) and IP_3_, thereby coupling TREM2 ligation to intracellular Ca^2+^ mobilization and protein kinase C activation (46) (**Figure 3**). This arm of the cascade feeds into nuclear factor of activated T-cells (NFAT) and nuclear factor kappa-B (NF-κB) transcriptional programs, but also directly reshapes membrane phosphoinositide pools that control phagocytic cup formation, endolysosomal trafficking, and lipid handling (49). Consistent with this, loss-of-function variants in PLCG2 phenocopy key aspects of TREM2 deficiency in human induced pluripotent stem cells (iPSC)-derived microglia exposed to myelin: both TREM2- and PLCγ2-deficient cells accumulate cholesterol esters, sulfatides, phospholipids, and triacylglycerols, indicating a shared defect in lipid catabolism and cholesterol efflux (46). By contrast, the AD-protective PLCG2 P522R “hypermorph” enhances PIP_2_ turnover and appears to bolster microglial resilience (73, 74), reinforcing the notion that the TREM2-DAP12-PLCγ2 axis is a key determinant of how microglia adapt to sustained lipid overload in the AD brain.

These proximal events converge on broader transcriptional and metabolic reprogramming. Downstream TREM2 signaling promotes metabolic and transcriptional reprogramming, including glycolytic adaptation, lysosomal biogenesis, and lipid-handling programs (**Figure 3**). These changes are characteristic of plaque-associated DAM-like states, whose transcriptional programs are strongly influenced by TREM2 dosage (22). Single-cell and spatial transcriptomic studies consistently show that many genes defining DAM, including *APOE*, lipoprotein lipase (*LPL*), cystatin F (*Cst7*), and numerous lysosomal and lipid transport genes, are sensitive to TREM2 dosage, implying that the receptor does not simply trigger an acute activation event but rather sets the trajectory of microglial state transitions under chronic stress (22).

Importantly, TREM2 signaling is not purely activating. Work in myeloid cells has highlighted an “ITAMi” mode in which low-affinity or partial ligation induces incomplete ITAM phosphorylation, preferentially recruiting phosphatases such as SHP-1 and dampening downstream activation (64). In the AD context, such tunable signaling may help explain how microglia integrate competing cues from TREM2 ligands (e.g., phosphatidylserine, APOE-containing lipoproteins, Aβ aggregates) with signals from other receptors, including toll-like receptors (TLRs), complement receptors, TAM family phosphatidylserine receptors, and inhibitory receptors such as CD33 (30) (**Figure 3**). Crosstalk with CSF1R signaling is particularly notable: CSF1R-driven Src activity can prime DAP12 ITAMs, thereby lowering the threshold for TREM2-dependent responses, whereas TREM2 signaling in turn feeds back on CSF1R-regulated survival and proliferation pathways (75). In this view, TREM2 acts less as a binary on-off switch and more as a rheostat that calibrates microglial responsiveness to a complex and evolving extracellular milieu (50).

Proteolytic processing of TREM2 adds yet another regulatory layer. Shedding of the ectodomain by ADAM10/17 at H157 produces soluble TREM2 (sTREM2) and leaves behind a C-terminal fragment that is subsequently cleaved by γ-secretase (56). Shedding terminates signaling by the full-length receptor and may deplete TREM2-DAP12 complexes from the surface, but the released ectodomain is not inert. sTREM2 can bind Aβ assemblies, influence plaque morphology, and has been reported to elicit ERK1/2 activation and cytokine production in neighboring microglia effectively acting as a paracrine amplifier of microglial activation (76). The net effect of enhanced shedding is therefore difficult to predict *a priori*: AD-risk variants such as H157Y that increase cleavage may simultaneously weaken membrane TREM2 signaling and augment sTREM2-mediated effects, with potentially divergent consequences for plaque dynamics, tau spreading, and synaptic integrity (35).

These signaling layers help explain how TREM2 supports adaptive microglial responses under early or limited stress but may become insufficient under chronic Aβ, tau, and lipid burden, particularly in the setting of hypomorphic variants (46, 54, 77) (**Figure 3**). In this sense, TREM2-associated signaling provides a mechanistic basis for stage-dependent microglial state transitions in AD.

## 4 Microglial development, homeostasis, and TREM2 in steady state

Microglia arise from primitive erythromyeloid progenitors in the yolk sac, which invade the neuroepithelium early in embryogenesis and subsequently establish a long-lived, self-renewing population that persists throughout life (23). Their developmental trajectory is marked by successive transcriptional waves that accompany transitions from proliferative embryonic microglia to postnatal pre-microglia and ultimately to mature homeostatic microglia (78). These stages are demarcated by dynamic regulation of lineage-determining transcription factors, including PU.1, IRF8, MEF2 family members, MAFB, and Sall1, which orchestrate microglial identity, metabolic competence, and responsiveness to tissue-derived cues (79). The acquisition of adult homeostatic signatures is tightly linked to sustained TGF-β and CSF1R signaling, both of which maintain survival and suppress aberrant inflammatory activation under physiological conditions (80, 81).

In the adult brain, microglia continuously survey their environment with highly motile processes, guided by neuronal and glial signals such as CX3CL1-CX3CR1, ATP-P2RY12, and the complement system (24, 82). The homeostatic gene module, including P2RY12, TMEM119, CX3CR1, G protein-coupled receptor 34 (GPR34), and transforming growth factor beta receptor 1 (TGFBR1), encodes receptors and signaling molecules that tune microglial responsiveness to subtle fluctuations in neuronal activity, synaptic remodeling, and myelin turnover (19). Within this framework, TREM2 is expressed at moderate basal levels and functions as a permissive regulator of microglial stability rather than a dominant driver of activation. Under steady-state conditions, ligand availability is limited mainly to low-abundance phospholipids, lipoproteins, and apoptotic cell, derived signals, resulting in a tonic, low-intensity TREM2-DAP12/DAP10 signaling baseline. Although subtle, this signaling contributes to maintenance of metabolic homeostasis and survival pathways, including PI3K-AKT and SYK-dependent cytoskeletal dynamics (51). Microglia lacking TREM2 exhibit a heightened sensitivity to environmental stressors, reduced survival in nutrient-limited conditions, and impaired basal phagocytic clearance of myelin debris and apoptotic cells, functional deficits that become detectable even in the absence of overt pathology (83).

Emerging data suggest that TREM2 also influences how microglia process endogenous lipids under non-pathological conditions. Lipidomic analyses indicate that tonic TREM2 activity supports cholesterol efflux and lysosomal lipid catabolism, preventing accumulation of cholesterol esters and oxidized lipids that otherwise predispose microglia toward a dysfunctional, lipid-droplet-rich phenotype (54). This is consistent with observations across multiple studies that TREM2-deficient microglia progressively accumulate intracellular lipids even in homeostasis, an early echo of the pronounced metabolic dysregulation seen under amyloid or myelin stress (21).

Importantly, steady-state TREM2 activity appears to maintain microglia in a poised, responsive configuration. Rather than being quiescent, homeostatic microglia operate near a threshold between surveillance and activation, and TREM2 serves as one of the molecular gatekeepers that calibrate the transition (84). Thus, understanding TREM2 in the steady state is essential not only for appreciating its role in pathology but also for recognizing its function as a molecular rheostat that sets the sensitivity of microglia to tissue perturbations.

Given this dual nature, therapeutically targeting TREM2 requires careful attention to its steady-state functions. Global inhibition risks destabilizing microglial homeostasis, compromising their trophic interactions with neurons and reducing resilience to age-related stress (49). As our understanding deepens, steady-state TREM2 emerges not merely as a passive background receptor but as a foundational regulator that shapes how microglia interpret environmental cues and determines the trajectory of microglial state transitions in health and disease.

## 5 DAM and TREM2-dependent microglial state transitions

The discovery of microglial heterogeneity through single-cell transcriptomics has fundamentally altered the prevailing concept of microglia as a homogeneous population that merely reacts to amyloid deposition (19). Instead, microglia undergo a structured and hierarchical transition in response to amyloid stress, marked by profound rewiring of transcriptional, metabolic, and functional programs. Central to this transition is TREM2, which enables microglia to adapt to chronic proteopathic stress (22).

Early during amyloid accumulation, microglia undergo a destabilization of their homeostatic identity. Characteristic markers involved in process motility, neuron-microglia communication, and purinergic signaling, such as P2RY12, TMEM119, and CX3CR1, are progressively downregulated as microglia approach areas enriched in oligomeric Aβ (22). This “stage-1” shift reflects the dissolution of the transcriptional program maintained by TGF-β and core lineage factors and represents a poised intermediate state rather than an inflammatory activation per se (85). At this stage, microglia undergo subtle remodeling of lipid metabolism, cytoskeletal dynamics, and lysosomal capacity, effectively priming them for the more dramatic reprogramming that will follow.

**Figure 4:**
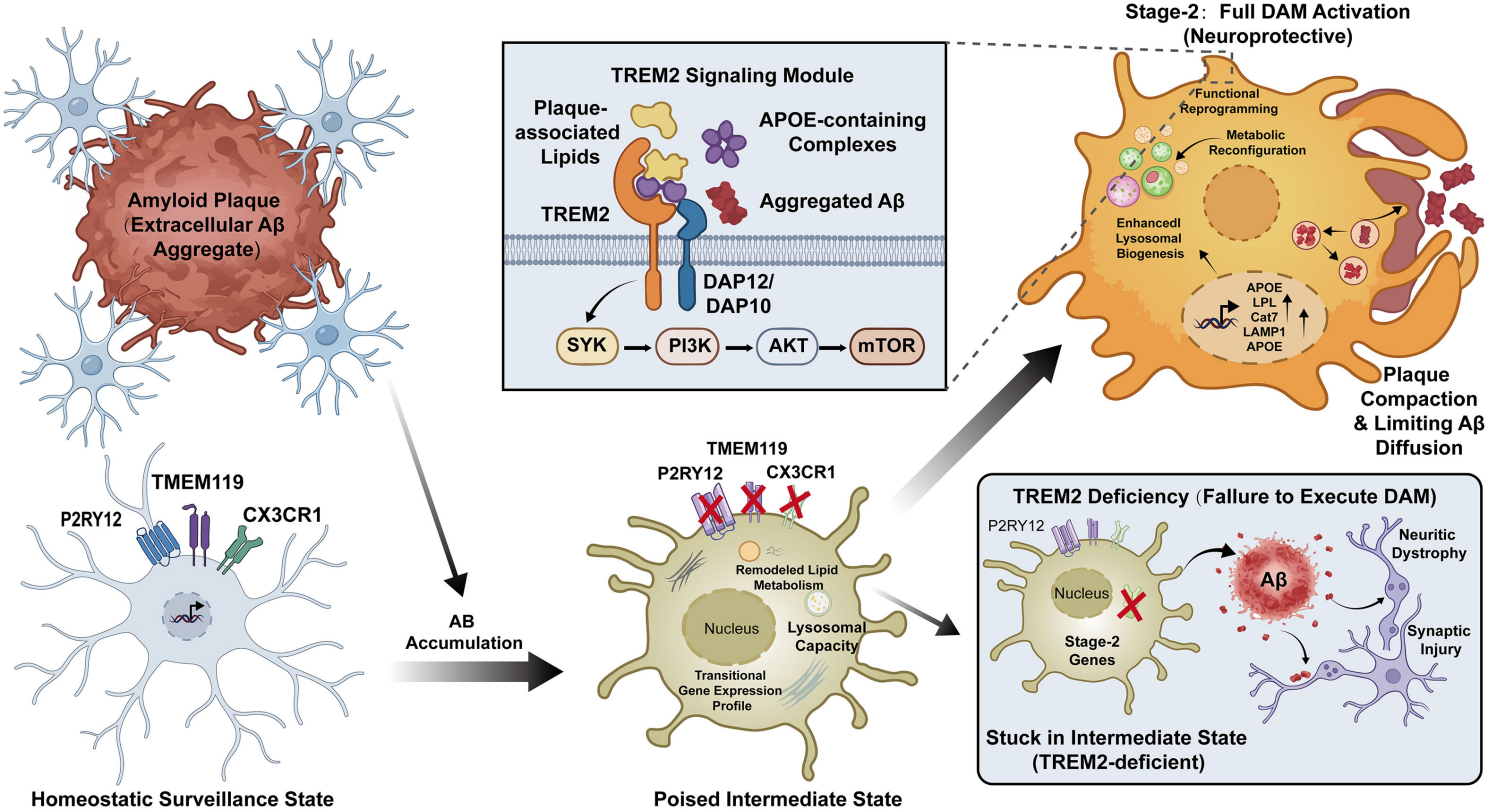
TREM2-dependent microglial state transitions in response to amyloid-β stress. Schematic illustrating the progression of microglia from a homeostatic surveillance state, characterized by expression of P2RY12, TMEM119, and CX3CR1, through an early poised intermediate state and into fully activated stage-2 disease-associated microglia (DAM) in the vicinity of amyloid-β plaques. In this framework, TREM2 functions as a key molecular checkpoint that enables the transition from the intermediate state to the plaque-associated DAM phenotype, accompanied by loss of homeostatic features, acquisition of phagocytic and stress-adaptive programs, and assembly of a compact microglial barrier around plaques. When TREM2 signaling is impaired, microglia fail to complete this state transition, remain arrested in an intermediate state, and are associated with poorly compacted plaques and increased neuritic and synaptic injury.

The full execution of the DAM program, often referred to as “stage-2 DAM”, requires intact TREM2 signaling and is illustrated in **Figure 4** primarily as a transcriptional and phenotypic state transition rather than a detailed metabolic pathway model (51) (**Figure 4**). Engagement of TREM2 by plaque-associated signals activates downstream pathways that support DAM-associated metabolic and lysosomal adaptation (42, 48) (**Figure 3**). This signaling axis supports the induction of a transcriptional module enriched for genes involved in phagocytosis, lipid processing, and antigen presentation, including *APOE*, *LPL*, *Cst7*, lysosomal-associated membrane protein 1 (*LAMP1*), and multiple cathepsins (20, 22). In parallel, microglia migrate toward plaques and assemble into a compact barrier that reorganizes plaque topology and limits the outward diffusion of neurotoxic Aβ species (77, 86).

TREM2 deficiency disrupts each of these steps. Without TREM2, microglia linger in the stage-1 intermediate and fail to activate the full DAM transcriptional network. Their inability to induce key metabolic and lysosomal genes leaves plaques poorly organized and more diffusely distributed, a structural defect associated with heightened neuritic dystrophy and synaptic injury (51). The protective elements of DAM, plaque compaction, sequestration of toxic Aβ conformers, and metabolic adaptation, are therefore inseparable from TREM2’s role in enabling microglial state transitions. Although DAM is often enriched for inflammatory mediators, the net functional outcome of TREM2-dependent DAM formation in early-to-mid disease stages appears to be neuroprotective (86).

Beyond the canonical DAM pathway, amyloid-bearing tissue elicits additional microglial states whose emergence depends on disease stage, tissue microenvironment, and TREM2 availability (87). Among these, interferon-responsive microglia, major histocompatibility complex class II (MHC-II)-enriched microglia, and proliferative microglial clusters represent parallel adaptive branches that may complement or compete with DAM (88). Several studies show that TREM2 modulates the distribution and intensity of these states. For example, TREM2 insufficiency dampens the expansion of phagocytic and lipid-metabolic microglia, while TREM2 agonism increases proliferative and antigen-presentation modules (89, 90). These observations suggest that TREM2 does not drive a single fixed microglial phenotype but rather shapes the topology of microglial activation trajectories within the diseased brain.

Human AD tissue reinforces the notion of conserved TREM2-dependent microglial remodeling, albeit with variations in magnitude and composition. Single-nucleus sequencing studies reveal microglial subclusters enriched for APOE, TREM2, lysosomal genes, and lipid metabolism signatures, elements reminiscent of DAM but embedded within a more heterogeneous cellular landscape (52). These findings illustrate that human microglia undergo a slower, more gradual transition compared to the sharp bifurcations found in mouse models, which may be due to variations in aging, chronicity, and cellular environment. Nevertheless, the shared reliance on TREM2 as a molecular checkpoint underscores its centrality in microglial adaptation to amyloid pathology across species.

Viewed together, these findings support a model in which TREM2-dependent state transitions constitute a core arm of the microglial response to AD (**Figure 4**). As AD progresses, however, the initially adaptive DAM program may become compromised or insufficient, raising the possibility that therapeutic modulation of TREM2 could strengthen or recalibrate microglial trajectories during critical windows of disease evolution (50). Understanding how TREM2 orchestrates these transitions provides a mechanistic framework that unifies animal model data with human observations and highlights microglial state modulation as a promising therapeutic frontier. However, the extent to which these TREM2-dependent state transitions are conserved across mouse models and human AD remains incompletely resolved, and current human evidence is still largely associative rather than mechanistically causal.

## 6 TREM2 in lipid metabolism, cholesterol handling, and myelin dynamics

**Figure 5:**
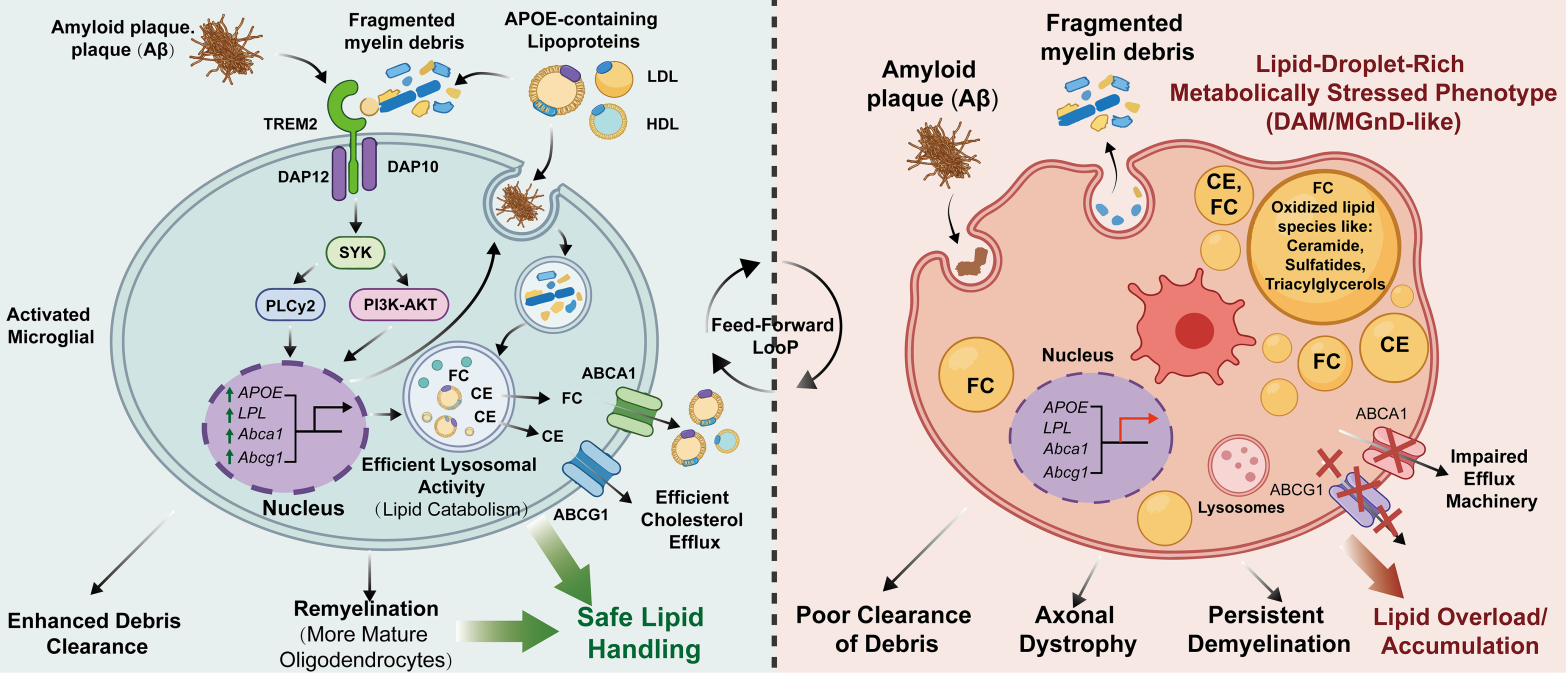
TREM2-mediated microglial lipid metabolism and myelin remodeling. Schematic highlighting the downstream metabolic and lipid-processing functions of TREM2 signaling in microglia exposed to chronic phagocytic stress from amyloid plaques, myelin debris, and lipoprotein-associated cargo. Through DAP12/DAP10-PLCγ2-SYK-PI3K-AKT-linked pathways, intact TREM2 signaling coordinates lipid uptake with lysosomal processing, cholesterol trafficking, APOE/LPL-associated lipid handling, and cholesterol efflux programs such as ABCA1 and ABCG1, thereby promoting efficient debris clearance and remyelination. By contrast, impaired TREM2 or PLCγ2 signaling leads to defective lipid processing, accumulation of cholesteryl esters and oxidized lipids in cytoplasmic lipid droplets, metabolic stress, and persistence of plaque- and myelin-associated pathology.

Beyond regulating microglial state transitions, TREM2 also supports the metabolic adaptation required for microglia to cope with chronic lipid burden, phagocytic stress, and myelin-derived debris in the diseased brain. Microglia exposed to persistent phagocytic stress, as in amyloid plaque, rich cortex or in demyelinating white matter, are forced to process large quantities of cholesterol, sphingolipids, and phospholipids. Under these conditions, TREM2 links recognition of lipid-rich cargo to downstream programs that support phagocytosis, lysosomal function, and lipid handling (40, 42, 48). Together, these findings highlight the metabolic dimension of TREM2 signaling in microglia, particularly in cholesterol handling, lipid catabolism, and myelin debris processing (**Figure 5**).

In the amyloid-laden cortex, plaque-associated microglia profoundly remodel their lipid metabolism. Single-cell and bulk transcriptomic analyses show robust induction of genes required for uptake, intracellular trafficking, and export of cholesterol, including *APOE*, *LPL*, and ATP-binding cassette transporters such as *Abca1* and *Abcg1* (40). TREM2 signaling is a major driver of this induction: in its absence, microglia still encounter and engulf Aβ and membrane fragments, but they fail to fully engage the efflux machinery, leading to a disproportionate storage of cholesterol as cholesteryl esters and the accumulation of oxidized lipid species (55). The lipidomic studies of human iPSC-derived microglia are particularly instructive in this regard. When challenged with myelin, TREM2- or PLCγ2-deficient microglia display convergent increases in cholesteryl esters, free cholesterol, ceramides, sulfatides, and triacylglycerols, indicating that disruption of the TREM2-DAP12-PLCγ2 axis compromises the cell’s ability to maintain cholesterol balance under lipid overload (46).

The consequences of impaired TREM2 signaling are even more striking in models of demyelination, where myelin-derived cholesterol constitutes the dominant lipid burden. In cuprizone (CPZ)-treated mice, TREM2-deficient microglia exhibit a dramatic increase in cholesteryl esters and oxidized derivatives compared with wild-type microglia, despite similar or even increased uptake of myelin fragments (55). Intact TREM2 signaling facilitates the efflux of excess cholesterol, likely through APOE-containing lipoprotein particles, thereby preventing the conversion of free cholesterol into storage esters. In TREM2-null settings, this efflux is inefficient, and microglia remain loaded with myelin-derived lipids, which translates into poor clearance of debris, fewer mature oligodendrocytes, axonal dystrophy, and persistent demyelination (83). Similar conclusions emerge from broader demyelination-focused reviews, which emphasize that microglial failure to clear myelin and recycle cholesterol is a key barrier to effective remyelination and that TREM2 is one of the principal receptors that licenses this clearance response (63). Together, these findings suggest that TREM2 shapes not only microglial behavior in plaque-rich gray matter but also the trajectory of white matter repair.

The intimate link between TREM2 and lipid metabolism is further underscored by its interaction with APOE. TREM2 binds APOE-containing lipoproteins with high affinity (41), and TREM2 activation, in turn, promotes microglial upregulation of APOE and LPL, forming a feed-forward loop that enhances lipid uptake and processing (25). In AD models, this TREM2-APOE axis underlies the transition from a homeostatic microglial phenotype to a DAM/MGnD-like state, which is characterized by coordinated changes in lipid metabolism, phagocytosis, and inflammatory gene expression (25). Importantly, APOE itself is a major genetic risk factor for LOAD, and isoform-specific differences in lipid packaging and receptor engagement likely influence how TREM2-positive microglia cope with cholesterol-rich substrates in the diseased brain (30). From this perspective, the combined genetic risk conferred by APOE4 and TREM2 variants could reflect a quantitative failure of the same pathway: insufficient capacity to package, export, and redistribute cholesterol and other lipids that accumulate as plaques and myelin deteriorate (**Figure 5**).

These mechanistic insights reposition TREM2 as a central regulator of myelin dynamics rather than a receptor acting exclusively in the vicinity of amyloid plaques. By facilitating efficient removal of damaged myelin and routing of liberated cholesterol into export pathways, TREM2-positive microglia create a microenvironment that is permissive for oligodendrocyte progenitor cell differentiation and remyelination (55). In TREM2-deficient mice, myelin debris persists longer in white matter tracts and remyelination is delayed or incomplete, linking TREM2 dysfunction to chronic white matter injury (83). Clinically, the demyelinating and leukoencephalopathic features of PLOSL and related TREM2/DAP12 mutation syndromes provide independent genetic support for a role of this pathway in maintaining myelin and bone integrity (32, 91). In sporadic AD and mixed dementia, where vascular lesions and white matter abnormalities are common, impaired TREM2-driven lipid handling in microglia could therefore amplify the contribution of small-vessel disease and leukoaraiosis to cognitive decline (51).

Viewed in aggregate, these data support a model in which TREM2 acts as a metabolic rheostat that couples recognition of lipid-rich danger signals to the microglial machinery for cholesterol efflux, lipid recycling, and myelin repair (**Figure 5**). When this signaling is compromised, by rare coding variants, reduced expression, or overwhelming lipid burden, microglia transition toward a lipid-laden, oxidatively stressed, and functionally impaired state that is less capable of restraining pathology. Therapeutically, this view suggests that enhancing TREM2 signaling or its downstream lipid-handling pathways could be a promising strategy to bolster microglial resilience, particularly in early disease stages when remodeling of cholesterol metabolism and myelin is still plastic (50). At the same time, the tight coupling between TREM2 activity, APOE biology, and systemic lipid metabolism cautions that interventions will need to be precisely tuned to avoid tipping microglia into chronically overactivated or metabolically exhausted states. Although the link between TREM2 and microglial lipid handling is supported by substantial experimental evidence, whether these metabolic alterations are primary drivers of AD progression or secondary adaptations to ongoing tissue damage remains unresolved.

## 7 sTREM2: biogenesis, biomarker value, and functional roles

The functional consequences of TREM2 signaling in AD are further modified by receptor shedding and genetic variation, which together influence ligand engagement, downstream signaling strength, and microglial responses across disease stages. sTREM2 arises from tightly regulated proteolytic processing of membrane-anchored TREM2, a mechanism that functions as both a molecular rheostat for receptor availability and a means of generating a bioactive extracellular fragment (56). The shedding event occurs within the stalk region, where α-secretases such as ADAM10 and ADAM17 cleave the ectodomain, a process clearly delineated in mechanistic studies describing H157 as the principal scissile position (92). After ectodomain release, the residual C-terminal fragment undergoes γ-secretase-mediated intramembrane proteolysis, terminating signaling (56, 93). Yet this process is not merely degradative. Multiple studies emphasize that cleavage efficiency is shaped by ligand occupancy, microenvironmental lipid composition, and receptor conformation, suggesting that shedding is coupled to the metabolic and inflammatory milieu in which microglia operate (57). The observation that disease-associated variants near the cleavage site, most notably H157Y, alter the rate of ectodomain release underscores the sensitivity of this system to subtle structural perturbations, and positions sTREM2 generation as an additional axis through which AD-linked alleles modulate microglial behavior (35).

In human biofluids, sTREM2 is measurable in both CSF and plasma, with CSF concentrations showing a more robust dynamic range (94, 95). Longitudinal studies synthesized across the retrieved literature converge on the view that sTREM2 increases early in the AD continuum, rising during preclinical and prodromal stages and reaching maximal levels near symptom onset (96, 97). Its temporal pattern tracks more closely with markers of neuronal injury, such as phosphorylated tau and neurofilament light chain, than with Aβ, supporting the interpretation that sTREM2 indexes microglial activation and neuroaxonal stress rather than amyloid deposition per se (59, 98). In this sense, sTREM2 does not simply mirror microglial density; instead, it reflects a specific activation state in which microglia transition toward a damage-responsive phenotype (57).

An intriguing feature consistently noted across multiple cohorts is that higher CSF sTREM2 in mildly symptomatic individuals associates with slower cognitive decline (58, 99). This suggests that sTREM2 may index a more efficacious or resilient microglial response during a critical window of disease progression, in line with transcriptomic evidence showing that microglial activation states associated with TREM2 signaling support amyloid compaction, clearance, and metabolic stabilization. The biomarker’s predictive value thus appears to derive not from signaling impending degeneration, but from marking the degree of microglial engagement with pathological stimuli (58, 100).

Beyond its utility as a biomarker, sTREM2 itself appears to possess biological activity. Although the precise receptor or interaction partner mediating its effects remains undefined, experimental studies report that extracellular sTREM2 enhances microglial survival, promotes proliferation, and increases metabolic activity under tissue-stress conditions (60). These actions conceptually align with the idea that sTREM2 serves as a paracrine amplifier of microglial responsiveness, complementing the membrane-bound receptor by extending TREM2 signaling into a broader spatial domain. Work in mouse models further supports this notion: exogenous delivery or overexpression of sTREM2 reduces amyloid burden, increases microglial clustering around plaques, and improves behavioral performance, effects consistent with enhanced microglial recruitment and phagocytic engagement (76, 101). In these contexts, sTREM2 functions not merely as a cleavage byproduct but as a facilitator of microglial transition toward a disease-associated state capable of structural remodeling and debris clearance.

However, accumulating observations suggest that sTREM2’s actions are context-dependent. Its capacity to stimulate inflammatory cytokine release could be beneficial in initiating containment responses but detrimental when chronically sustained (60). Given that microglial phenotypes in AD arise from a delicate interplay between lipid metabolism, phagocytosis, and immune signaling, prolonged elevations in sTREM2 may skew microglia toward maladaptive activation states that contribute to synaptic loss or propagate tau pathology (61, 101). This duality mirrors the broader complexity of TREM2 biology, in which the same molecular pathways that facilitate neuroprotection early in disease may exacerbate degeneration when regulatory boundaries are lost. Thus, therapeutic strategies centered on modulating sTREM2, or enhancing or suppressing TREM2 shedding, must consider the timing, magnitude, and inflammatory context in which sTREM2 is manipulated (57).

**Figure 6:**
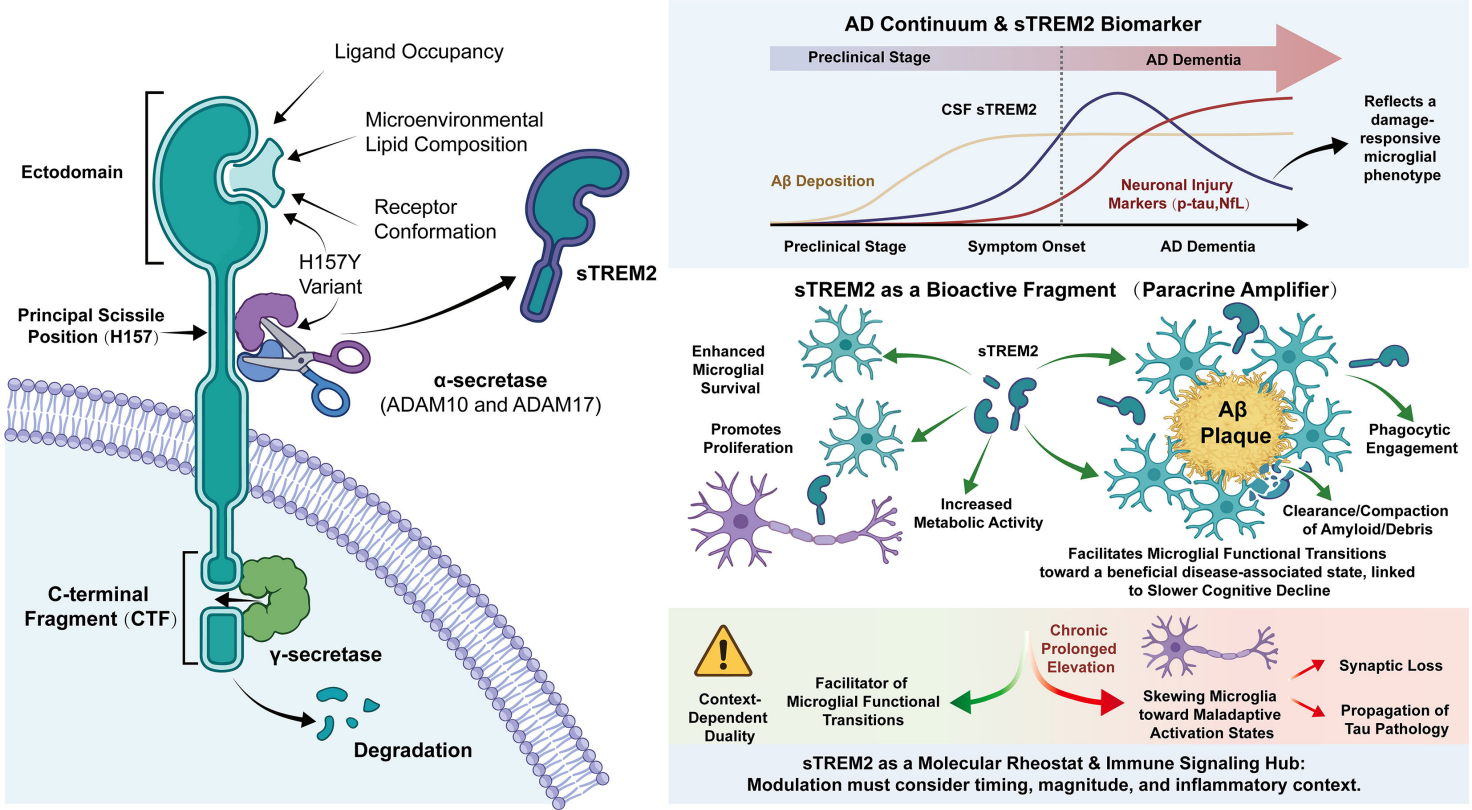
Biogenesis, biomarker dynamics, and context-dependent actions of soluble TREM2 in AD. Schematic of TREM2 ectodomain shedding from microglial membranes, in which ADAM10/17 cleave the stalk region at H157 to release sTREM2 and leave a C-terminal fragment that is subsequently processed by γ-secretase, with shedding efficiency modulated by ligand occupancy, lipid milieu, receptor conformation, and disease-associated variants such as H157Y. In human CSF, sTREM2 levels rise early along the AD continuum and track markers of neuroaxonal injury rather than Aβ load, indexing TREM2-dependent microglial state transitions. Extracellular sTREM2 acts as a paracrine mediator that can enhance microglial survival, proliferation, metabolic activity, and clustering around plaques to promote amyloid clearance, but chronic elevation may drive maladaptive activation, synaptic injury, and tau propagation, underscoring its role as a molecular rheostat within the TREM2 immune signaling hub.

Taken together, sTREM2 emerges as a multifaceted component of the TREM2 signaling architecture: a dynamically regulated product of receptor processing, a sensitive biomarker of microglial state transitions, and a bioactive mediator that shapes microglial function in both protective and potentially harmful directions (57) (**Figure 6**). Understanding when sTREM2 signifies adaptation versus maladaptation will be essential for leveraging it not only as a readout of microglial activity but also as a candidate therapeutic axis in its own right.

## 8 Human TREM2 variants and clinical phenotypes

Because TREM2 integrates extracellular sensing, microglial state control, and metabolic fitness, it has become a therapeutically actionable node rather than merely an observational marker of disease-associated microglial activation. Human genetics provides a graded view of how perturbations in the TREM2-DAP12 axis translate into human disease (**Table 2**). At the severe end of this spectrum, biallelic loss-of-function mutations in TREM2 or its signaling adaptor DAP12 cause PLOSL, a disorder in which progressive cognitive decline, striking white matter degeneration, and cystic bone lesions coexist (32). Clinically, these patients often present with behavioral or frontal lobe syndromes in young adulthood, followed by rapidly progressive dementia; histopathology reveals frontotemporal atrophy, demyelination, and grossly abnormal microglia, indicating that complete disruption of TREM2-DAP12 signaling destabilizes both CNS myelin maintenance and bone remodeling (91). Notably, some homozygous TREM2 mutations that were first identified in PLOSL pedigrees can also produce frontotemporal dementia, like syndromes without overt skeletal involvement (102), suggesting that genetic background and modifier factors shape phenotypic expression.

**Table 2:** Human TREM2 variants and associated clinical, mechanistic, and biomarker phenotypes.

Variant class/Representative mutations	Species	Model system	Genetic context	Clinical phenotype	Mechanistic consequences	CSF/Biomarker features	Notes/Modifiers
Biallelic loss-of-function (PLOSL/Nasu-Hakola disease) *e.g*., null alleles in *TREM2* or *DAP12* (*TYROBP*)	Human; mouse (supporting studies)	Patients with PLOSL/Nasu-Hakola disease; TREM2- or Tyrobp-deficient mouse models	Homozygous or compound heterozygous	Early-onset dementia with behavioral/frontal symptoms; Severe leukoencephalopathy, white matter degeneration; Cystic bone lesions	Near-complete disruption of TREM2-DAP12 signaling; Failed microglial activation; abnormal microglial morphology; Impaired CNS myelin maintenance and bone remodeling	Very low CSF sTREM2 due to defective folding/trafficking and absent regulated shedding	Genetic background influences presence/absence of skeletal involvement; Same alleles may produce FTD-like syndromes without bone disease
AD-associated hypomorphic missense variants (LOAD risk alleles) *R47H*, *R62H*, *H157Y*, others	Human; mouse; iPSC-derived microglia	AD patient cohorts; knock-in mouse models; human iPSC-microglia systems	Heterozygous	Increased risk for late-onset AD (R47H: OR ~2–4 in Northern Europeans); Some increase in risk for FTD, DLB; less consistent links to ALS/PD	Partial loss of ligand binding (R47H/R62H): reduced affinity for APOE, anionic lipids, Aβ; Dampened DAM induction; reduced plaque compaction; impaired chemotaxis; H157Y: increased ectodomain shedding → altered balance of membrane vs soluble TREM2	R47H: often elevated CSF sTREM2 (stage-dependent); H157Y: increased sTREM2 due to accelerated shedding	Strong ancestry dependence (R47H effect attenuated outside Northern Europe); Variants shift microglial activation threshold, not complete LOF
Protein-folding/trafficking-disrupting variants (often PLOSL-linked)	Human; cell-based systems; mouse (selected studies)	Patients carrying rare TREM2 variants; heterologous expression systems; selected transgenic/knockout models	Homozygous or heterozygous (depending on variant)	PLOSL-like or mixed phenotypes; May present with dementia without bone cysts	Mutations cluster in buried regions → impaired folding, ER retention, loss of surface expression; Functionally severe LOF	Low CSF sTREM2 due to failure to reach membrane	Phenotypic severity modified by environment/co-modifier loci
Variants near the proteolytic cleavage site *H157Y* and related	Human; cell-based systems; mouse (selected studies)	AD cohorts; *in vitro* shedding assays; knock-in or expression models	Heterozygous	Typically associated with AD or mild neurodegenerative phenotypes	Increased ADAM-mediated shedding → high sTREM2 but functional insufficiency in DAM transition	Elevated CSF sTREM2	Increased shedding ≠ protection; qualitative signaling defects persist
Rare variants in non-AD neurodegenerative diseases *e.g., R47H*, others in FTD, DLB, PD, ALS	Human; mouse; iPSC-derived microglia	Disease-specific patient cohorts; proteinopathy mouse models; iPSC-based disease models	Heterozygous	Modest or inconsistent increases in disease risk; Disease-specific regional vulnerability	Impaired microglial responses to tau, α-synuclein, TDP-43; Failure to adopt appropriate microglial states around diverse proteinopathies	Variable; often similar to AD-associated alleles	Suggests TREM2-regulated microglial resilience as a shared mechanism across diseases
General pattern across variants (continuum model)	Human; mouse; *in vitro* systems	Integrated interpretation across clinical, genetic, and experimental studies	–	Spectrum from severe leukodystrophy → primary dementias → mild AD-risk alleles	Null alleles = collapse of immunometabolic microglial function; Hypomorphic AD-risk alleles = increased activation threshold; Cleavage-site variants = altered soluble vs membrane signaling balance	sTREM2 as dynamic, allele- and stage-dependent biomarker	APOE4 strongly modifies R47H phenotype; TREM2-APOE axis crucial for DAM transition and plaque engagement

In contrast, the risk architecture of LOAD is dominated by heterozygous missense variants that shift TREM2 function more subtly. The R47H substitution remains the best-studied example: in individuals of Northern European ancestry, it increases the odds of AD by approximately two- to four-fold (33, 103), comparable to the effect of a single APOE ϵ4 allele, whereas its impact appears attenuated or absent in several non-European populations, underscoring strong ancestry dependence (104). Structural and biochemical work indicates that R47H and related AD-associated substitutions reside on the exposed surface of the Ig-like domain, where they weaken interactions with anionic phospholipids, apolipoproteins such as APOE, and aggregated Aβ, without fully abolishing ligand recognition (105). By contrast, many Nasu-Hakola variants cluster in buried regions of the protein, destabilizing folding and trafficking (106). Together, these observations support a continuum model in which AD-linked alleles behave as hypomorphic variants that preserve some signaling competence but raise the threshold for microglial activation around plaques and sites of tissue damage, whereas PLOSL mutations sit at the extreme of near-complete pathway failure.

The clinical expression of TREM2 risk variants is further modulated by interactions with other major genetic factors, particularly APOE. Carriers of both APOE4 and R47H tend to exhibit earlier symptom onset and more aggressive clinical courses than carriers of either variant alone (107), and neuropathological series suggest that this combination is associated with a higher probability of meeting criteria for definite AD at autopsy (105). At the same time, individuals harboring R47H in the absence of APOE4 can still develop typical AD pathology, indicating that TREM2 dysfunction is sufficient, in at least a subset of cases, to drive the full clinicopathological phenotype (107). These gene-gene interactions dovetail with experimental data showing that TREM2 and APOE cooperate to establish the DAM state: when this axis is compromised, plaque-associated containment and lipid handling are impaired (25). From a mechanistic standpoint, human variant data therefore argue that clinical risk is not simply a function of amyloid burden, but also of how efficiently microglia, under the influence of TREM2 and APOE, transition into, and sustain, adaptive response states in the face of ongoing pathology.

Functional studies of knock-in mice and iPSC-derived human microglia carrying R47H, R62H, H157Y, or other AD-associated TREM2 variants reinforce this view. Across models, these alleles consistently impair DAM-like responses and plaque-associated microglial barrier functions, with worsening neuritic dystrophy and synapse loss (69, 108). R47H knock-in animals, for example, display impaired chemotaxis toward ATP gradients, reduced capacity to compact Aβ deposits, and a propensity to accumulate lipid droplets and cholesteryl esters when challenged with myelin debris, phenotypes that echo, in milder form, the profound defects seen in TREM2 or TYROBP null mutants (109). Variants near the proteolytic cleavage site, such as H157Y, add an additional layer of complexity by altering the balance between membrane-bound and sTREM2: they accelerate ectodomain shedding and raise CSF sTREM2 concentrations, yet still behave as risk alleles (35, 70). This pattern suggests that higher sTREM2 is not necessarily protective, and that variant effects depend on how TREM2 signaling is redistributed between membrane-bound and soluble forms.

Human fluid biomarker studies provide an important bridge between genotype and phenotype. Carriers of classical Nasu-Hakola-associated mutations tend to have markedly reduced CSF sTREM2 levels, consistent with failure of properly folded receptor to reach the cell surface and undergo regulated shedding (94), whereas carriers of R47H or H157Y often exhibit elevated CSF sTREM2 (70). These opposing effects on sTREM2 highlight that TREM2 variants cannot be cleanly divided into “loss-of-function” and “gain-of-function” categories; instead, each allele produces a distinct pattern of receptor expression, shedding, and downstream signaling (57). Clinically, higher sTREM2 at the mild cognitive impairment or very early dementia stage often associates with slower cognitive decline and reduced structural atrophy (58, 99), implying that, at least transiently, robust TREM2-dependent microglial activation can buffer against accumulating pathology.

Finally, TREM2 variants are not restricted to AD and PLOSL. R47H and other missense substitutions have been reported at increased frequency in subsets of patients with frontotemporal dementia, dementia with Lewy bodies, and, more controversially, amyotrophic lateral sclerosis and Parkinson’s disease (36). Although effect sizes are generally smaller and replication inconsistent across studies, these observations suggest that TREM2-controlled microglial programs constitute a shared resilience mechanism engaged in response to diverse proteinopathies. When this axis is compromised, microglia may fail to execute the appropriate state transitions needed to contain tau, α-synuclein, or TDP-43 pathology in vulnerable networks, thereby accelerating neurodegeneration in a region- and disease-specific manner. From this perspective, “Human TREM2 variants and clinical phenotypes” can be viewed as natural experiments in tuning the microglial response curve: from near-complete pathway failure to subtler shifts in disease-related response states (110). Understanding how each class of variant reshapes this landscape will be essential for designing TREM2-targeted therapies that restore, rather than indiscriminately amplify, microglial function in AD and related dementias.

## 9 TREM2 beyond AD: cross-disease perspectives

While the primary focus of this review is AD, the functional roles of TREM2 extend far beyond neurodegeneration, encompassing a range of systemic and metabolic conditions, including cancer, obesity, and non-alcoholic fatty liver disease (NAFLD). This expanding landscape underscores TREM2’s central role as a mediator of immune responses to chronic stress, lipid dysregulation, and tissue damage, marking it as a potential therapeutic target across diverse pathologies (62).

In metabolic disorders such as obesity and NAFLD, TREM2 plays a crucial role in regulating lipid-associated macrophages (LAMs) within adipose and liver tissues. Studies have shown that in obesity, TREM2-positive (TREM2+) macrophages aggregate around hypertrophic adipocytes, where they help modulate local inflammation and contribute to insulin sensitivity, thus maintaining metabolic balance (62). However, the role of TREM2 in these contexts is complex, with some studies suggesting that it may also exacerbate inflammation under certain conditions. For example, in diet-induced obesity models, TREM2-deficient mice show worsened insulin resistance (IR) and greater adipocyte hypertrophy, suggesting that TREM2’s signaling pathway may act protectively in maintaining peripheral metabolic health (62).

In the liver, TREM2’s role extends to modulating macrophage activity during chronic injury, including NAFLD. Here, TREM2+ macrophages are involved in repairing tissue damage and modulating metabolic processes. Notably, TREM2 expression in macrophages has been linked to the regulation of lipid metabolism, with TREM2-deficient macrophages impairing hepatocellular energy metabolism and promoting the accumulation of ceramides, which are associated with insulin resistance and hepatic steatosis (111). This extends TREM2 biology beyond the CNS and links its dysfunction to both metabolic and neurodegenerative disease contexts.

Beyond metabolic diseases, TREM2 has emerged as a key player in cancer, particularly in tumor-associated macrophages (TAMs) (112). TREM2 is upregulated in various cancers, including lung (110, 112), gastric (113), and liver (114) cancer, where it contributes to the establishment of an immunosuppressive tumor microenvironment. TREM2+ TAMs can inhibit anti-tumor T cell responses, thereby promoting tumor growth and progression (114). This immunosuppressive role has made TREM2 a potential target for cancer therapy, with some studies suggesting that depleting TREM2+ macrophages or blocking TREM2 signaling could enhance the efficacy of immune checkpoint inhibitors (115, 116).

The duality of TREM2’s roles, across metabolic disease, neurodegeneration, and cancer underscores the context dependence of its signaling. Therapeutically, this implies that TREM2 may need to be activated in some settings but inhibited in others, consistent with its broader role in macrophage biology across tissues. Given its involvement in processes such as lipid metabolism, inflammation, and tissue remodeling, TREM2 represents a promising therapeutic target, but its manipulation will require a clearer understanding of tissue- and disease-specific signaling.

## 10 Therapeutic targeting of TREM2 signaling in AD

**Figure 7:**
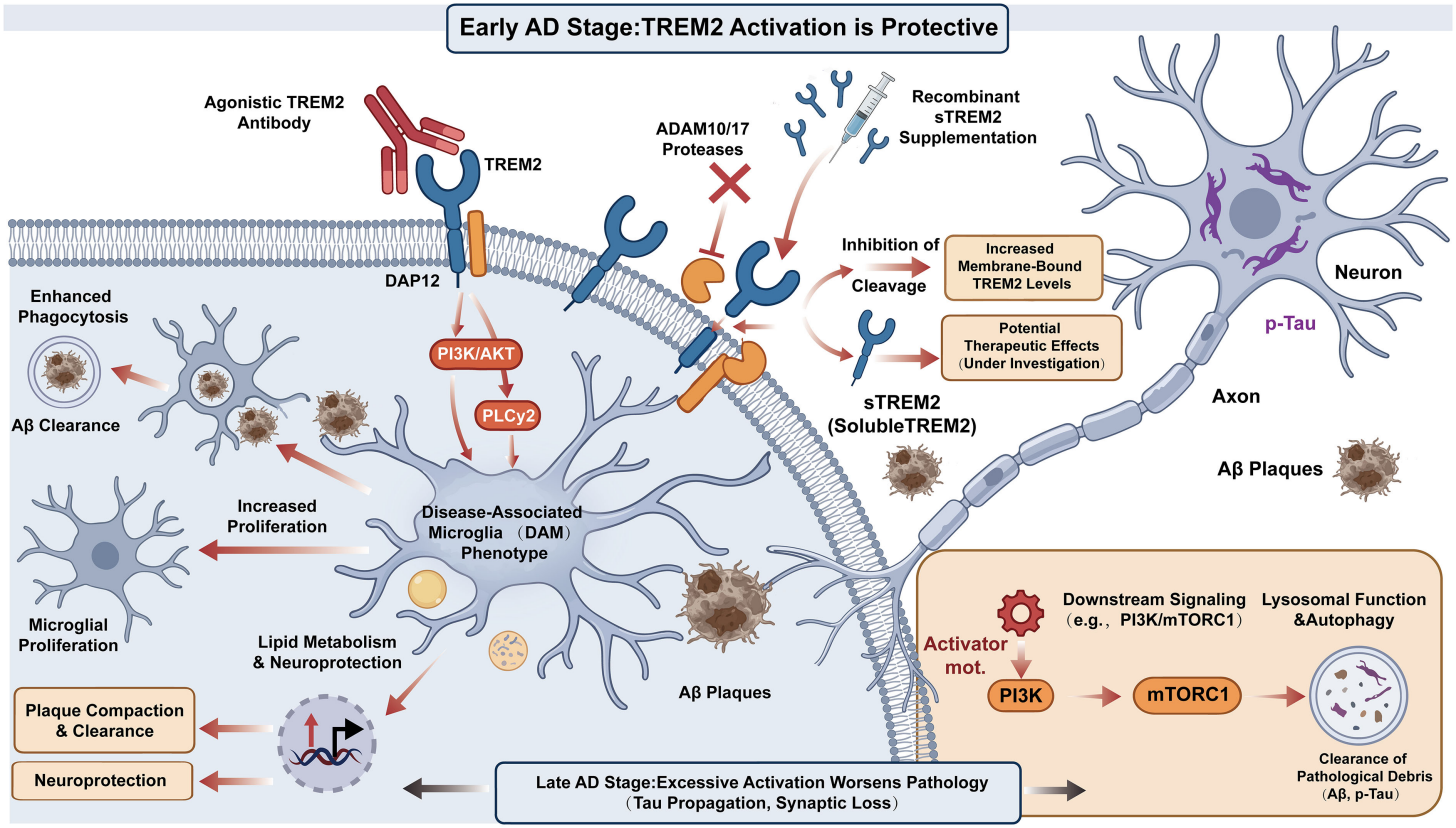
Therapeutic strategies targeting TREM2 signaling in AD. Schematic overview of microglia interacting with Aβ plaques and tau pathology, highlighting three main TREM2-focused interventions: (i) agonistic anti-TREM2 antibodies that enhance DAP12/DAP10-SYK-PI3K-PLCγ2 signaling, promote DAM-like activation, plaque compaction, and debris clearance; (ii) modulation of TREM2 shedding via ADAM10/17 or recombinant sTREM2 to tune the balance between membrane-bound TREM2 signaling and bioactive sTREM2; and (iii) targeting downstream nodes such as PI3K-mTORC1 to bolster lysosomal function and autophagy. The diagram emphasizes that the efficacy and safety of these approaches are stage- and context-dependent, with TREM2 activation being largely protective in early AD but potentially deleterious under chronic, late-stage microglial overactivation.

The increasing recognition of TREM2 as a pivotal modulator of microglial responses in AD has significantly advanced our understanding of potential therapeutic strategies. Unlike traditional approaches that focus primarily on the neuronal components of AD, such as Aβ and tau, TREM2-based therapies aim to enhance the immune functions of microglia. These innate immune cells play a central role in maintaining tissue homeostasis and regulating neuroinflammation. However, in AD, microglial function becomes dysregulated, exacerbating disease progression. The therapeutic manipulation of TREM2 signaling thus offers a novel strategy to not only modulate microglial activation but also to influence the broader neuroinflammatory environment that underpins AD pathology (**Figure 7**).

### 10.1 TREM2 agonistic antibodies and microglial activation

A promising strategy involves the use of agonistic monoclonal antibodies to enhance TREM2 signaling. Preclinical studies have shown that activating TREM2 with such antibodies can lead to increased microglial proliferation and a shift towards a DAM phenotype, promote plaque-associated microglial responses, and improve pathological and functional readouts in AD models (90). Clinical evaluation of TREM2-targeted agonistic antibodies has now progressed beyond early proof-of-concept studies, but the negative Phase 2 outcome of AL002 underscores the difficulty of translating target engagement into measurable clinical benefit in AD.

Among TREM2 agonistic antibodies, AL002 is the most clinically advanced example to date. AL002 is a humanized monoclonal antibody developed to activate TREM2 signaling on microglia, and preclinical studies showed evidence of target engagement, enhanced microglial proliferation, and reduced amyloid-associated pathology in AD mouse models. However, despite this encouraging preclinical profile, the Phase 2 INVOKE-2 trial in patients with early AD failed to meet its primary endpoint of slowing clinical decline, and no significant treatment effects were observed across secondary clinical, functional, or biomarker measures, despite evidence of sustained target engagement. The long-term extension study was subsequently terminated. These findings highlight an important translational challenge for TREM2-directed therapy: engagement of the receptor and induction of microglial activation may not be sufficient, by themselves, to produce meaningful clinical benefit, particularly if treatment timing, disease stage, or patient selection are suboptimal.

While these therapies have shown promise, several challenges remain. For instance, TREM2’s effects on microglial state transitions are context-dependent. In early stages of AD, enhancing TREM2 signaling could prove beneficial by promoting plaque containment and lipid clearance. Therapeutic effects are likely to be stage- and context-dependent: early enhancement of TREM2 may support plaque containment and lipid clearance, whereas persistent activation in later or tau-enriched settings may be less favorable (86). The AL002 experience further suggests that target engagement alone may be insufficient without appropriate biological and temporal matching (86) (**Figure 1**). This effect may partly relate to impaired microglial Aβ clearance in late-stage AD (117). Thus, fine-tuning the temporal activation of TREM2 is critical to avoid exacerbating pathological processes.

### 10.2 Modulating TREM2 shedding and sTREM2 levels

Another therapeutic avenue involves modulating the balance between membrane-bound TREM2 and its soluble form, sTREM2. This approach leverages the fact that TREM2 undergoes proteolytic cleavage by ADAM proteases, a process that releases sTREM2 into the extracellular space (56, 92). Elevated levels of sTREM2 in CSF have been associated with early stages of AD, and it is thought to reflect microglial activation (59). Therefore, strategies that increase the shedding of TREM2 or enhance sTREM2 production could serve as both a diagnostic biomarker and a therapeutic tool.

Inhibition of ADAM10/17, the proteases responsible for TREM2 cleavage, has been proposed as a way to enhance membrane-bound TREM2 levels, which may amplify microglial phagocytosis and inflammatory resolution. However, inhibiting these enzymes could have off-target effects, as ADAMs cleave numerous other substrates. More selective approaches, such as antibodies that block the cleavage site or stabilize TREM2’s membrane-bound form, might offer safer and more targeted interventions (35). Conversely, in situations where boosting the effects of sTREM2 is desirable, such as when promoting neuroprotection in the early stages of AD, therapies aimed at enhancing sTREM2 shedding or supplementing recombinant sTREM2 might be beneficial (76). However, given the context-dependent effects of sTREM2, such approaches would require biomarker-guided use (60).

### 10.3 Targeting downstream signaling nodes

While TREM2 itself is an upstream modulator of several signaling pathways, targeting its downstream signaling components may provide an alternative therapeutic strategy when direct receptor modulation is insufficient or genetically constrained. Importantly, this strategy is supported by experimental evidence: in TREM2-R47H mice, systemic anti-CLEC7A antibody treatment rescued microglial activation by engaging downstream SYK signaling, providing proof of concept for bypassing impaired TREM2-dependent responses (49). Beyond this SYK-linked rescue approach, other downstream effectors, including PI3K and PLCγ2, remain mechanistically attractive candidates because of their roles in microglial metabolic adaptation, phagocytosis, and inflammatory tuning (46, 48).

mTORC1 is another candidate downstream node of interest because of its established role in lysosomal function, autophagy, and microglial metabolic fitness, all of which are relevant to the clearance of neurotoxic material (51). However, the therapeutic manipulation of these pathways will require careful attention to cell specificity, disease stage, and systemic safety, as many of these signaling modules are broadly active in immune and non-immune cells beyond microglia.

### 10.4 Stage- and context-dependent considerations

A recurring theme in TREM2 biology is the importance of context and disease stage in determining the effects of therapeutic interventions (**Figure 1**). In broad terms, early activation may be more favorable than chronic activation in advanced or tau-enriched disease states (28). Thus, it is crucial that TREM2-targeted therapies be tailored to the stage of disease progression.

Additionally, individual genetic background factors, such as the APOE genotype, may influence how TREM2-based therapies perform in different patient populations (30). Personalized strategies that take into account these genetic and pathological variables will likely be necessary to optimize therapeutic outcomes.

Overall, TREM2 modulation has substantial therapeutic potential in AD. Its successful application will depend on a deeper understanding of microglial state transitions, lipid metabolism, and the molecular mechanisms governing TREM2 signaling. Future research should focus on defining the optimal timing, dosage, and patient stratification for TREM2-targeted therapies to ensure both efficacy and safety in treating AD.

### 10.5 Conflicting evidence and the boundary between protective and maladaptive TREM2 responses

The central controversy is not whether TREM2 matters, but when and in whom TREM2-driven programs are beneficial versus harmful (21, 50, 110). A dominant interpretation from amyloid-centric models is that TREM2 activation is protective, because it promotes plaque compaction, lipid buffering, and microglial metabolic fitness (51, 54, 77). However, this interpretation may be overgeneralized: many of these readouts are proximal biological effects rather than hard evidence of sustained neuroprotection across disease stages (4, 9). Thus, the key unresolved issue is whether early containment biology translates into durable systems-level benefit.

A competing body of work-particularly in tau-enriched or chronic settings-suggests that persistent DAM-like activation can coincide with synaptic stress, reduced homeostatic support, and propagation of neurodegenerative cascades (87, 108, 118). Importantly, this does not simply refute the protective model; rather, it supports a phase-shift hypothesis: the same TREM2-linked response may be adaptive during acute amyloid stress but maladaptive when activation becomes prolonged or biologically exhausted (19, 85, 88). In other words, apparent contradictions across studies may reflect differences in disease phase, pathology mixture, and baseline microglial state, rather than mutually exclusive biology (21, 87, 89).

Methodological asymmetry likely amplifies this conflict. Preclinical studies often infer benefit from histological or transcriptional endpoints, whereas clinical relevance depends on longitudinal cognitive outcomes; these are not equivalent evidence tiers (4, 9, 10). Likewise, model systems differ in ligand landscape and inflammatory tone, increasing the risk that context-specific conclusions are interpreted as universal rules (27, 52). Accordingly, the field should be cautious about universal claims that more TREM2 activation is beneficial across all stages (21, 88).

This debate has immediate translational consequences. Anti-TREM2 therapeutic development has not yet yielded definitive efficacy evidence in early AD, despite strong biological rationale, indicating that target engagement alone is insufficient without correct temporal and biological matching (9, 110). A stronger framework is a precision, stage-aware model: TREM2 modulation should be deployed according to disease timing, dominant pathology, and patient-level modifiers, and evaluated with longitudinal biomarkers that can detect both adaptive gain and maladaptive drift (63, 89, 97, 99). Under this framework, conflicting findings are not noise-they are boundary conditions that define where TREM2-directed therapy is likely to help, fail, or cause unintended effects.

## 11 Outstanding questions and future directions

Despite the rapid expansion of work on TREM2, the field still lacks a coherent model of how this receptor integrates diverse tissue cues into specific microglial states across the AD continuum. Future work should also investigate when TREM2-independent microglial programs predominate, and whether TREM2 is a driver, modifier, or marker across different AD stages. One major unresolved issue is the hierarchy and context-dependence of TREM2 ligands *in vivo*. Phosphatidylserine on apoptotic cells, myelin-derived lipids, APOE-containing lipoproteins, and Aβ-lipid complexes can all engage TREM2 (38, 41, 42), likely varies by brain region, disease stage, and comorbidity context. Current structural and biochemical data define ligand classes but not the *in situ* “ligand landscape” that microglia actually experience in human AD brains (65). Future work will need to combine lipidomics, spatial proteomics, and ligand-selective TREM2 variants or antibodies, ideally in models that recapitulate human APOE isoforms and white-matter injury, to map how local lipid composition, demyelination, and vascular leakage shape TREM2 engagement over time.

A second conceptual gap concerns the temporal logic and reversibility of TREM2-dependent microglial programs. Single-cell studies have established that TREM2 is a key driver of DAM and related activation trajectories, including lipid-droplet-rich and white-matter-associated states that emerge in response to amyloid, tau, and myelin damage (22, 54). Yet it remains unclear whether these phenotypes represent discrete, quasi-stable attractor states or flexible, reversible configurations along a continuum of stress responses. This uncertainty has direct therapeutic implications: a TREM2 agonist administered early might promote plaque-engulfing states, whereas the same intervention at later stages, could reinforce chronically activated or maladaptive microglial states (86). Longitudinal single-cell and spatial multi-omics in mouse models and human tissue, ideally anchored to patient-level clinical and biomarker trajectories, will be required to resolve whether TREM2-driven programs can be re-set and, if so, at which points in the disease course intervention is still reversible.

Human relevance remains another central challenge. Mouse studies have been indispensable for defining TREM2 pathways, but interspecies differences in baseline microglial states, ligand exposure, and lifespan complicate extrapolation (15). Adult human microglia exhibit pronounced regional and developmental heterogeneity, with subsets in subventricular zones, thalamus, and association cortex displaying distinct activation markers and proliferative profiles even in the absence of overt pathology (19). At the same time, population genetics has revealed substantial ancestry- and variant-specific heterogeneity in TREM2-associated risk, with R47H, R62H, H157Y and other substitutions conferring different effect sizes and being present at markedly different frequencies across European, Asian, and African-derived cohorts (104, 119). These observations argue strongly against a one-size-fits-all model of TREM2 function. Humanized mouse strains, microglia-containing organoids, and ex vivo slice systems that incorporate human TREM2 haplotypes, APOE isoforms, and mixed cellular milieus will be essential to understand how specific genetic backgrounds and regional microenvironments constrain or reshape TREM2-dependent state transitions.

In parallel, it is increasingly evident that TREM2 does not operate in isolation but sits within a densely interconnected genetic network that includes APOE, CD33, INPP5D/SHIP1, PLCG2, ABCA7, and other microglia-enriched AD risk genes. Epistatic and pathway-level interactions between these loci are only beginning to be explored. For example, APOE is required for full acquisition of the DAM phenotype and functions as a major TREM2 ligand (25, 30), while CD33 and INPP5D/SHIP1 can antagonize ITAM signaling and thereby dampen TREM2-DAP12 cascades (120). Variants in PLCG2 and TYROBP further modulate the strength and quality of downstream calcium and metabolic signaling (46, 121). Moving forward, systems-level approaches that integrate genetics, phospho-signaling, metabolic flux, and chromatin accessibility will be needed to delineate how these pathways converge on shared transcriptional modules, and how different combinations of risk alleles steer microglia toward distinct, clinically relevant trajectories of plaque handling and neuronal support or injury.

The biology of sTREM2 presents another layer of complexity that remains incompletely defined. Proteolytic cleavage of membrane TREM2 and alternative splicing generate sTREM2 species that are measurable in CSF and plasma and show dynamic changes across preclinical and symptomatic stages of AD (57, 122). While rising CSF sTREM2 in early disease is often interpreted as a marker of microglial activation, experimental data suggest that soluble and membrane-bound TREM2 may have partly divergent or even opposing roles in microglial regulation and AD pathology (76, 100). Variants that alter TREM2 shedding, such as H157Y, further complicate interpretation of sTREM2 as a biomarker and as a therapeutic target (35, 70). Dissecting which cell types contribute to sTREM2 in different disease stages, how distinct sTREM2 isoforms signal, and how pharmacologic modulation of shedding impacts both receptor availability and downstream microglial states will be critical before strategies aimed at boosting or inhibiting sTREM2 can be rationally deployed.

Finally, translation of TREM2 biology into therapy will require a more nuanced integration of CNS and systemic physiology. TREM2 is not only a microglial receptor but also shapes lipid handling and inflammatory tone in peripheral macrophages, adipose tissue, and liver, where it contributes to obesity, atherosclerosis, and fatty liver disease, conditions that themselves modify AD risk and brain resilience (62, 111). This raises the possibility that TREM2-targeted interventions could have divergent effects in brain and periphery, and that baseline metabolic status will modulate both efficacy and safety. Moreover, preclinical work suggests that the net impact of enhancing TREM2 signaling is likely to depend on disease stage, sex, APOE genotype, and regional pathology (90). There is a pressing need for pharmacodynamic biomarkers that report TREM2 pathway engagement and microglial functional state *in vivo*, combining trajectories of CSF sTREM2, microglia-selective PET ligands, and blood or CSF transcriptomic/proteomic signatures, to guide dose selection, identify optimal treatment windows, and stratify patients according to TREM2-related risk architecture. Only by embedding TREM2 modulation within this broader, temporally and genetically informed framework will it be possible to determine whether targeting microglial state transitions can meaningfully alter the course of AD.

## 12 Concluding remarks

**Figure 8:**
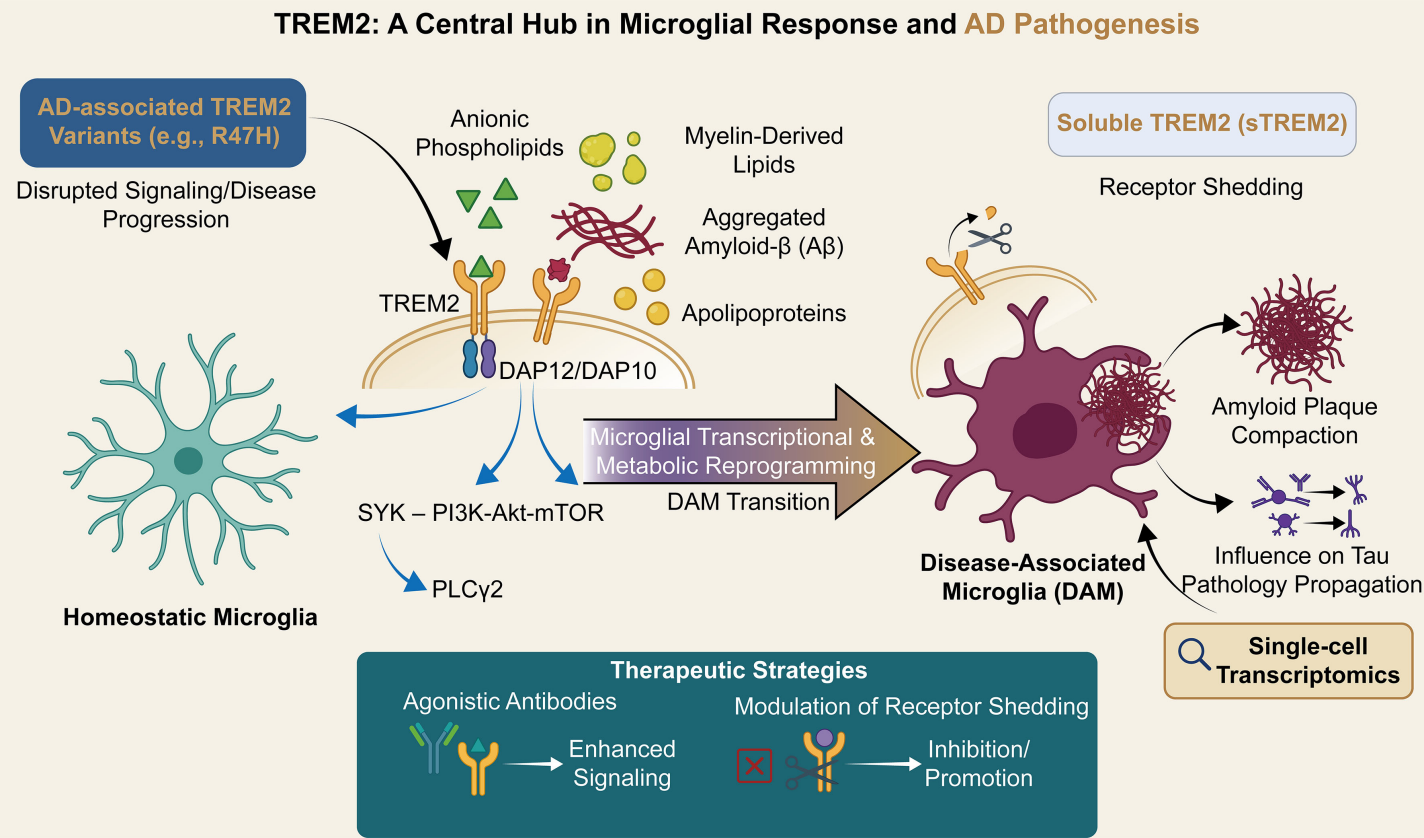
TREM2 acts as a central hub in microglial responses and AD pathogenesis.

TREM2 has emerged as a critical node in AD, connecting microglial activation, tissue damage, and lipid metabolism to the disease’s pathogenesis. The receptor’s role in modulating microglial responses, especially in its interaction with a variety of lipids and damage-associated molecular patterns, marks a significant departure from the traditional neuron-centric view of AD. Ligand binding, including phosphatidylserine, myelin debris, and aggregated Aβ, activates TREM2 signaling (38, 44). These pathways promote plaque-associated, DAM-like microglial states involved in phagocytosis, survival, and tissue homeostasis (48, 51) (**Figure 8**).

Genetic studies have strongly linked TREM2 variants, particularly the R47H mutation, to an increased risk of LOAD, underscoring the receptor’s role in neurodegeneration (33, 103). While these findings emphasize the importance of TREM2 in neuroinflammation, they also highlight the complexity of its function: partial loss of TREM2 function exacerbates disease risk, whereas complete loss of TREM2 causes early-onset dementia, as seen in PLOSL (32). This duality suggests that TREM2’s influence is highly context-dependent, influenced by the timing, intensity, and location of its activation.

However, TREM2-mediated microglial activation is not always beneficial. Inappropriate or prolonged activation may contribute to the progression of tau pathology and neuroinflammation (86, 110). For instance, early TREM2 activation could be beneficial, while late-stage activation may become maladaptive. Future research should aim to delineate the precise windows in which TREM2 signaling is protective versus deleterious, taking into account genetic, environmental, and pathological factors. In this Review, we synthesize these observations into a stage-resolved immunometabolic framework intended to guide when TREM2 modulation may be beneficial, neutral, or potentially harmful.

Furthermore, while TREM2 presents a promising target for therapeutic interventions, the challenges remain considerable. Its interaction with numerous ligands, including phospholipids and apolipoproteins, as well as its soluble form (sTREM2), complicates the development of therapeutic strategies (49, 57). Variations in TREM2 expression and function across populations, coupled with differences in the genetic backgrounds of patients, suggest that tailored approaches will be necessary (104). Studies into the effects of TREM2 on lipid metabolism both in the brain and peripherally may yield insights into how peripheral diseases such as hypercholesterolemia and atherosclerosis influence AD progression (62).

Ultimately, TREM2 has helped reshape current models in AD research, by highlighting the dynamic roles of microglia as both protectors and potential mediators of neurodegeneration. Its modulation presents exciting therapeutic opportunities, but the full therapeutic potential of TREM2 can only be realized through interdisciplinary research that integrates structural biology, immunology, genetics, and clinical neuroscience. Importantly, this framework supports stage-specific patient stratification and testable trial designs, rather than a uniform TREM2-targeting strategy across all phases of AD. Understanding the full scope of TREM2’s influence on microglial state transitions, lipid metabolism, and neuroinflammatory processes is essential for the development of safe, effective TREM2-targeted therapies in AD and other neurodegenerative disorders.
